# Taliglucerase Alfa Reduces Amyloid-β Burden by Restoring Autophagic Pathways in a Neuronal Model of Alzheimer’s Disease

**DOI:** 10.1007/s11064-026-04792-w

**Published:** 2026-06-01

**Authors:** Çağrı Özkurt, Selma Köse, Çimen Karasu, Arjan Kortholt, Pelin Kelicen-Uğur

**Affiliations:** 1 Department of Pharmacology, Faculty of Pharmacy, Hacettepe University, Ankara, Turkey; 2 Department of Pharmacology, Faculty of Pharmacy, Lokman Hekim University, Ankara, Turkey; 3 Department of Medical Pharmacology, Faculty of Medicine, Gazi University, Ankara, Turkey; 4 YETEM-Innovative Technologies Application and Research Centre, Suleyman Demirel University, Isparta, Turkey; 5 Department of Cell Biochemistry, University of Groningen, Groningen, The Netherlands

**Keywords:** Taliglucerase alfa, β-Glucocerebrosidase, Alzheimer’s disease, Autophagy, Lysosomal storage disorder, Enzyme replacement therapy

## Abstract

Intraneuronal amyloid-beta (Aβ) accumulation and autophagic dysfunction are key pathological features of Alzheimer’s disease (AD). Mutations in *GBA1*, which encodes the lysosomal enzyme β-glucocerebrosidase (GCase), are linked to several neurodegenerative disorders, but the role of GCase in AD remains incompletely understood. In this exploratory, proof-of-concept study, we investigated whether taliglucerase alfa (TAL), a recombinant human GCase, may influence intracellular Aβ accumulation by modulating autophagy pathways in a neuronal AD model. Endogenous Aβ accumulation was induced in mouse hippocampal neuronal cells (HT-22) by exposure to low-molecular-weight Aβ_1−42_ oligomer-enriched assemblies (oAβ_1−42_), followed by treatment with TAL. Soluble Aβ levels and selected components of the autophagy–lysosome pathway, including GCase, cathepsin B, p62/sequestosome-1 (p62/SQSTM1), and mammalian target of rapamycin (mTOR), were evaluated using Western blotting, ELISA, and RT-PCR. In this *in vitro* model, TAL treatment was associated with a reduction in intracellular monomeric Aβ levels. This observation was accompanied by changes in mTOR signaling and p62 levels, suggestive of modulation of autophagy-related processes. Overall, these results provide preliminary, hypothesis-generating evidence supporting a potential association between lysosomal GCase augmentation and Aβ-related and autophagy-associated processes in AD. Further studies, including expanded experimental validation and *in vivo* investigations, are required to clarify the underlying mechanisms and translational relevance.

**Supplementary Information:**

The online version contains supplementary material available at 10.1007/s11064-026-04792-w.

## Introduction

Alzheimer’s disease (AD) is the most prevalent neurodegenerative disorder, defined by the presence of extracellular senile plaques formed by amyloid-beta (Aβ) and intracellular neurofibrillary tangles (NFTs) composed of hyperphosphorylated tau protein. While the extracellular aggregation of Aβ is central to the onset and progression of AD [1]; recent studies suggest that the intraneuronal accumulation of Aβ, driven by oligomer internalization, is neurotoxic and may play a critical role in advancing the disease [2–4].

Glucocerebrosidase (GCase) is a lysosomal enzyme responsible for catalyzing the hydrolysis of glucosylceramide into glucose and ceramide. Deficiencies in GCase, encoded by the *GBA1* gene, have been linked to various neurodegenerative diseases. Homozygous mutations in *GBA1* are specifically associated with Gaucher’s disease (GD), an inherited lysosomal storage disorder [5, 6]. GD patients typically express less than 15% of functional GCase, which causes accumulation of glucosylceramide and glucosylsphingosine in the lysosomes, leading to cellular damage and pathological events in the spleen, bone marrow, liver, lungs, and brain [7]. Taliglucerase alfa (TAL), a recombinant human glucocerebrosidase analogue (rhGCase or rhGBA), is produced in genetically modified carrot cells by recombinant DNA technology [8]. This plant-based expression system naturally targets the enzyme to the plant vacuole (a lysosome-like organelle), resulting in glycoproteins that possess the desired terminal mannose residues without requiring the post-production enzymatic modifications needed for enzymes produced in mammalian cell lines [9]. Native GCase is targeted from the Golgi apparatus to the lysosome via an intracellular pathway mediated by the lysosomal integral membrane protein type-2 (LIMP-2), a process confirmed to be independent of the mannose-6-phosphate receptor system [9, 10]. TAL, marketed in the U.S. as ELELYSO^®^ (Pfizer) for injection is indicated for the treatment of patients 4 years and older with confirmed diagnosis of Type 1 GD. It is a monomeric glycoprotein enzyme containing four N-linked glycosylation sites (MW: 60.8 kDa). It differs from native human GCase by two amino acids at the N terminal and up to 7 amino acids at the C terminal. TAL is a glycosylated protein with oligosaccharide chains at the glycosylation sites having terminal mannose sugars. These mannose-terminated oligosaccharide chains are a key design feature, as they are specifically recognized by cell-surface carbohydrate receptors, principally the mannose receptor (MR). This mechanism facilitates the efficient endocytosis of the exogenous enzyme, a process distinct from the intracellular trafficking of native GCase, which relies on the LIMP-2 escort protein [8, 10]. The involvement of GCase in the pathogenesis of AD remains largely underexplored. Recently, it was demonstrated that GCase expression and enzyme activity in the brain of AD patients is lowered and that this deficiency could play a role in the development of AD by inducing lysosomal dysfunction. In addition, it was demonstrated that GCase lentivirus (human complementary DNA of *GBA1*) facilitates the clearance of Aβ_1−42_ oligomers and protects against Aβ_1−42_ oligomer-induced neuronal cell death by enhancing lysosomal function [11].

Lysosomes collaborate with autophagosomes to degrade and recycle misfolded cytoplasmic proteins, such as Aβ, and organelles within the cytoplasm [12, 13]. Increased deposition of Aβ is considered a key factor in the pathogenesis of AD [14], and impaired autophagy has been observed in both animal models of AD and AD patients [15–17]. Thus, strategies targeting autophagy-related proteins to regulate Aβ production, clearance, and aggregation hold significant potential for the treatment of AD [18]. Autophagy begins with the formation of preautophagosome (phagaphore) membrane which is formed from multiple membrane sources such as the endoplasmic reticulum, mitochondria, plasma membrane, and endosomes. The elongation of the phagaphore is driven by the activation of early-stage autophagy-related proteins (Atgs), including Atg5, Atg12, Atg14, Atg16L, and beclin-1. Soluble form of microtubule-associated protein 1 light chain 3-I (LC3-I) is a cytosolic Atg that undergoes post-translational modification into the membrane-bound form LC3-II during autophagosome formation [19], the phagaphore closes and matures into an autophagosome. This machinery facilitates the lipidation of LC3, which allows autophagy receptors like p62 to tether ubiquitinated cargo to the growing autophagosome for eventual degradation [20]. The mature autophagosome membrane fuses with the lysosomal membrane, activating late lysosomal proteins and presenting its contents to lysosomal enzymes (cathepsins B, D, and L) for degradation in the acidic lysosomal environment [21]. The formation of autophagosomes is also regulated by the mammalian target of rapamycin (mTOR). AMP-activated protein kinases (AMPK; heterotrimeric serine/threonine protein kinases) are also key regulators of body metabolism. It is well-established that AMPK activation induces autophagy by suppressing mTOR, the primary inhibitor of autophagosome formation. Studies have shown that AMPK activators enhance autophagy by inhibiting mTOR signaling and trigger Aβ degradation via the lysosomal system. Conversely, Aβ has been found to increase mTOR activity, while reductions in Aβ levels decrease mTOR activity, providing evidence of the reciprocal relationship between mTOR and Aβ [22, 23]. Furthermore, the levels of p62/sequestosome-1 (p62/*SQSTM1*), an autophagic cargo protein and a widely recognized marker of autophagic flux, accumulate during autophagy inhibition and diminish when autophagy is activated [24].

Additionally, sestrins are highly conserved proteins encoded by stress-responsive genes, such as those activated by DNA damage, oxidative stress, and hypoxia. Among the three sestrin isoforms expressed in mammalian cells (SESN1, SESN2, SESN3) [25], SESN2 has been the most extensively studied, with its cytoprotective effects attributed to its antioxidant activity and its role in inducing autophagy [26, 27]. SESN2, which is induced by stress conditions [28], promotes autophagy induction by activating AMPK and subsequently suppressing mTOR activity [29, 30].

To directly investigate the impact of augmenting functional GCase within neuronal lysosomes, we selected TAL for this study over other GCase-enhancing strategies such as small-molecule chaperones (e.g., ambroxol) or substrate-reduction agents (e.g., venglustat). This approach was chosen based on compelling evidence from other lysosomal storage disorders; for example, in Pompe disease, an enzyme replacement therapy (ERT) glycoengineered for enhanced M6P receptor binding demonstrated superior substrate clearance compared to approaches relying on chaperone co-administration [31]. As an ERT, TAL delivers fully active, well-characterized recombinant GCase, enabling a direct assessment of enzyme supplementation. Furthermore, TAL’s mannose-terminated glycans are designed to promote efficient cellular uptake and lysosomal delivery via mannose receptors, ensuring the enzyme reaches its intended site of action. The availability of GMP-grade TAL also provided high purity and batch-to-batch consistency, crucial for the reproducibility of our mechanistic *in vitro* investigations.

The present study hypothesized that TAL might restore GCase and protect autophagic function by improving lysosomal efficacy in neuronal cells exposed to oAβ_1−42_, potentially mitigating Aβ accumulation. To evaluate this hypothesis, we examined the impact of TAL on Aβ accumulation in mouse hippocampal neurons (HT-22 neuronal cells) exposed to low–molecular-weight Aβ_1−42_ oligomer-enriched assemblies (oAβ_1−42_), as well as key markers of the autophagy-lysosome pathway implicated in AD.

## Results

### Exposure of oAβ_1−42_ Increased Cytotoxicity, and TAL Treatment Partially Preserved MTT-Based Metabolic Activity HT-22 Cells from oAβ_1−42_ Toxicity

**Fig. 1:**
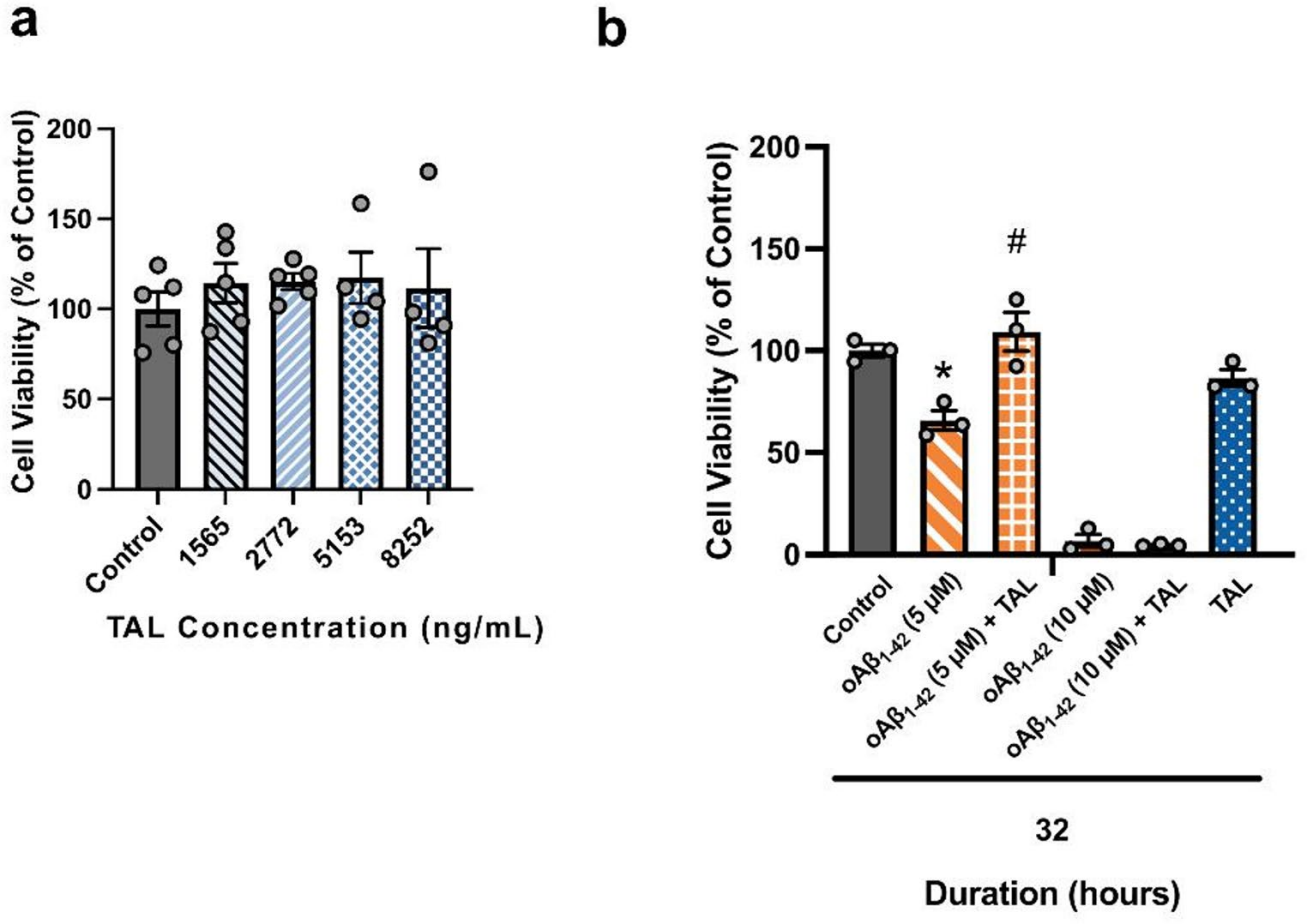
TAL protects HT-22 neurons from oAβ_1−42_-induced cytotoxicity at the selected 32-hour time point. Cell viability was assessed by MTT assay. Data are presented as mean ± SEM (*n* = 3–5), n indicates the number of independent biological experiments. **a** TAL was not cytotoxic at concentrations up to 8252 ng/mL after a 32-hour exposure. **b** The neuroprotective effect of TAL against oAβ_1−42_ was evaluated after a 32-hour incubation. This time point was chosen as it provided a clear window of cytotoxicity from 5 µM oAβ_1−42_ that was fully rescued by TAL (see Online Resource 1 Fig. S1 for 24- and 48-hour data). Endogenous GCase (GBA) protein expression was confirmed in HT-22 cells using immunoblotting of total RIPA whole-cell lysates (Fig. S2). Statistical analyses were performed using one-way ANOVA followed by Dunnett’s (**a**) or Tukey’s (**b**) multiple comparisons tests. Significance is denoted as *p* < 0.05. * indicates a significant reduction in viability in the oAβ₁₋₄₂ group compared to the Control group. # indicates a significant rescue of viability in the oAβ_1−42_ + TAL group compared to the oAβ_1−42_ alone group

Initially, the effective concentrations of TAL were determined based on concentration ranges reported in the literature. In humans, TAL is administered intravenously every two weeks, primarily targeting peripheral effects in GD, with a pediatric dose of 30 U/kg and an adult dose of 60 U/kg. After 38 weeks of treatment, the maximum plasma concentrations were reported as 1656 ± 1116 ng/mL in pediatric patients and 5153 ± 3099 ng/mL in adult patients [32]. In our study, cytotoxicity assays were performed using the lowest median, and highest concentrations within the selected range. TAL was not cytotoxic to HT-22 cells when used at these concentrations for 32 h (F_(4,18)_ = 0.3218, *p* = 0.8596; Fig. 1a), therefore we used the highest concentration of 8252 ng/mL for further investigations (*p* = 0.9165).

To establish the optimal conditions for our *in vitro* model, a time-course experiment was performed to assess oAβ_1−42_-induced cytotoxicity. After a 32-hour incubation, 5 µM oAβ_1−42_ significantly reduced cell viability (*p* = 0.0009), an effect that was completely reversed by co-treatment with TAL (*p* < 0.0001; Fig. 1b). While the higher concentration of 10 µM oAβ_1−42_ was also toxic at this time point, this effect was not rescued by TAL. The full results for the 24- and 48-hour time points are provided in the Online Resource 1 (Fig. S1). Based on these observations, subsequent experiments were conducted using 5 µM oAβ_1−42_, 8252 ng/mL TAL, and a 32-hour incubation period as the standardized experimental conditions.

The effects of TAL treatment alone on cell viability were also assessed. After 32 h of exposure, there was no significant difference in viability compared to the control group (Fig. 1a). A moderate increase in viability was observed at 24 h and a minor increase was seen at 48 h (Online Resource 1 Fig. S1).

### Treatment with TAL Decreased Intraneuronal Aβ Accumulation and Increased Lysosomal GCase and Cathepsin B Levels

**Fig. 2:**
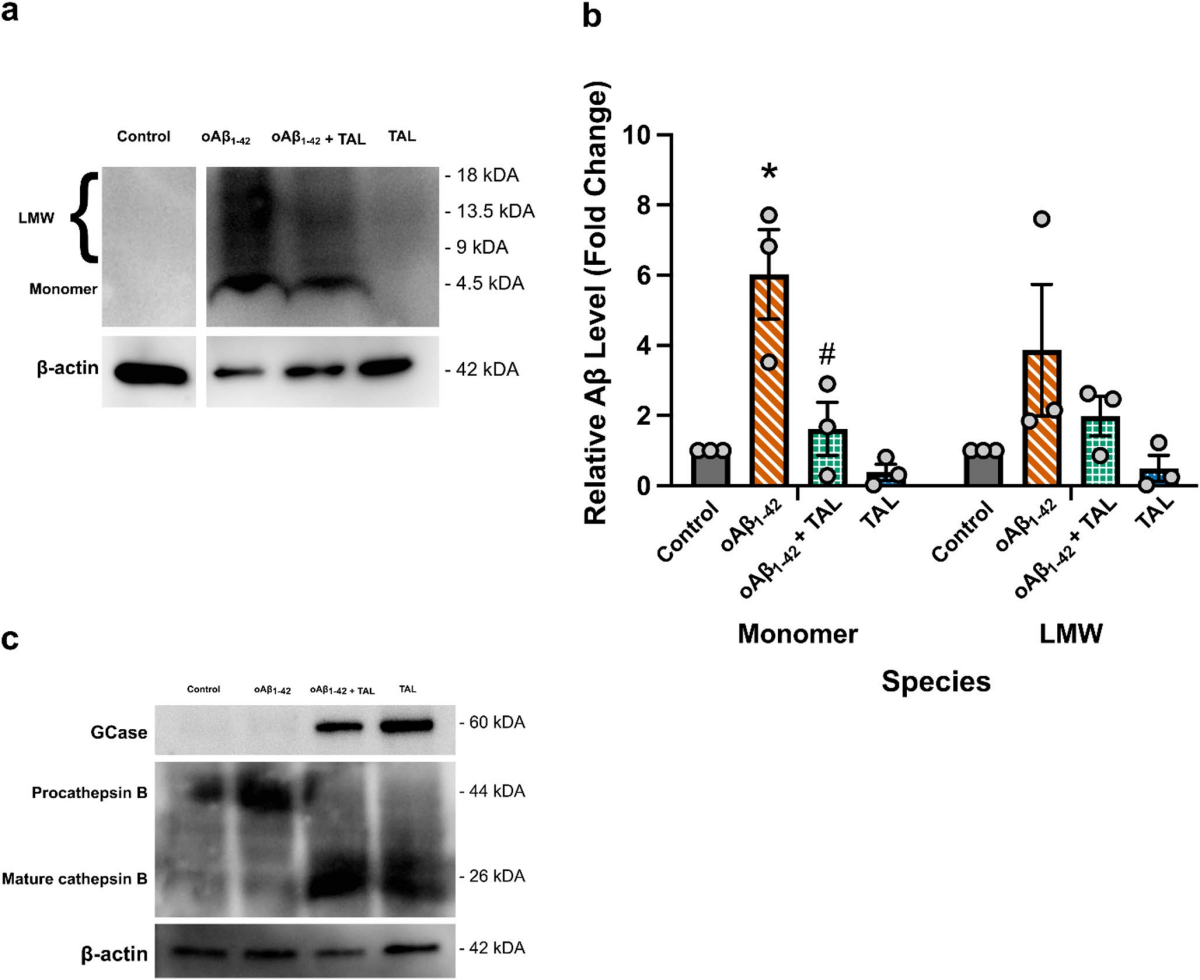
TAL selectively reduces intracellular monomeric Aβ and modulates lysosomal protein expression. HT-22 cells were treated for 32 h as indicated. **a** Representative Western blot showing intracellular levels of monomeric (4.5 kDa) and low-molecular-weight (LMW) oligomeric Aβ species. **b** Densitometric analysis of intracellular Aβ levels, normalized to β-actin and expressed as fold change relative to control. Data are presented as mean ± SEM (*n* = 3), n indicates the number of independent biological experiments. Statistical analysis was performed using a two-way ANOVA with Holm-Šídák’s multiple comparisons test. Significance is denoted as *p* < 0.05. * indicates a significant increase in monomeric Aβ in the oAβ_1−42_ group compared to the control group. # indicates a significant reduction in monomeric Aβ in the oAβ_1−42_ + TAL group compared to the oAβ_1−42_ alone group. **c** Representative Western blots from lysosome-enriched extracts showing the accumulation of GCase and the maturation of Cathepsin B in response to TAL treatment. All conditions were run on the same membrane. The uncropped full-length blot is provided in the Supplementary Raw Data file

Western blot analyses were conducted on HT-22 cell lysates following incubation with TAL and/or oAβ_1−42_. After 32 h of exposure to 5 µM oAβ_1−42_, a significant elevation was observed in monomeric Aβ (4.5 kDa) (*p* = 0.0038), while the increase in low molecular weight (LMW) Aβ forms—tetramers, trimers, and dimers—did not reach statistical significance (*p* = 0.0685; Fig. 2a, b). Co-treatment with TAL (8252 ng/mL) markedly attenuated the increase in monomeric Aβ levels (*p* = 0.0083), while levels of LMW Aβ forms remained unaffected (*p* = 0.1502; Fig. 2a, b).

Additionally, in lysosome-enriched extracts, oAβ_1−42_ treatment alone did not alter GCase protein expression relative to the control; however, co-treatment with oAβ_1−42_ and TAL, as well as TAL treatment alone, resulted in an accumulation of GCase protein in HT-22 cells (Fig. 2c). Notably, the lysosome-enriched extracts demonstrated the precursor (pro) form of cathepsin B in control and oAβ_1−42_-treated cells, in contrast to the detection of its active form following treatment with TAL, either independently or together with oAβ_1−42_ (Fig. 2c).

### TAL Modulates Key Proteins in the Autophagy Signaling Pathway

**Fig. 3:**
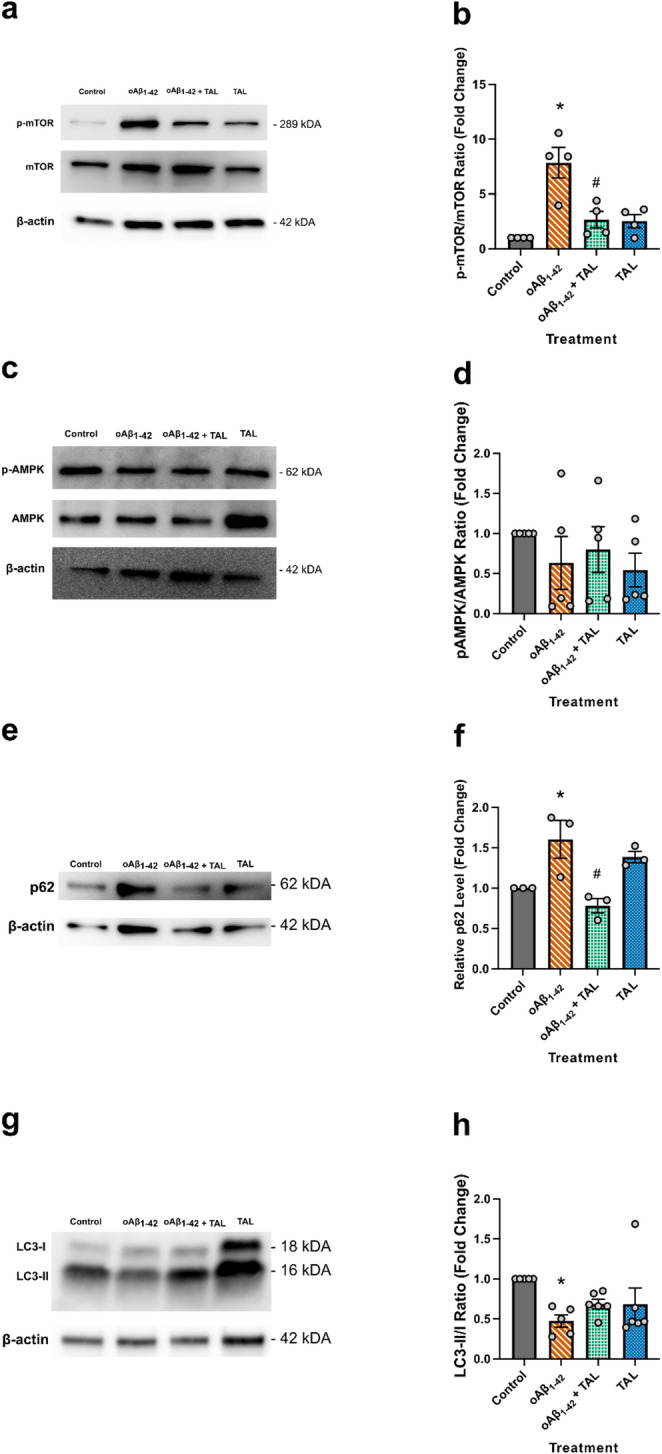
TAL modulates key proteins in the autophagy pathway dysregulated by oAβ_1−42_. HT-22 cells were treated for 32 h. Panels show representative Western blots (**a**, **c**, **e**, **g**) and corresponding densitometric analyses (**b**, **d**, **f**, **h**). Data are presented as mean ± SEM (*n* = 3–6) and expressed as fold change relative to control, n indicates the number of independent biological experiments. Statistical analysis was performed using one-way ANOVA with Tukey’s multiple comparisons test (*p* < 0.05). **a**, **b** oAβ_1−42_ significantly increased the p-mTOR/mTOR ratio (*), an effect reversed by TAL co-treatment (#). **c**, **d** No significant changes were observed in the p-AMPK/AMPK ratio among treatment groups. **e**, **f** p62 levels were significantly increased by oAβ_1−42_ (*), and this accumulation was reversed by TAL co-treatment. **g**, **h** While the overall one-way ANOVA for the LC3-II/I ratio did not reach statistical significance (F(3,18) = 2.906, *p* = 0.0630), post-hoc analysis revealed a significant decrease in the oAβ_1−42_ group compared to control (*). TAL co-treatment did not result in a significant rescue. In these panels, * denotes a significant difference in the oAβ_1−42_ group compared to the control group. # denotes a significant difference in the oAβ_1−42_ + TAL group compared to the oAβ_1−42_ alone group

To evaluate potential alterations in the autophagy pathway, we performed Western blot analyses on key regulatory proteins in extracts from HT-22 cells exposed to oAβ_1−42_ and/or TAL (Fig. 3).

First, we examined the phosphorylation of mTOR, a primary inhibitor of autophagy initiation. In HT-22 cells, oAβ_1−42_ exposure led to a significant increase in the p-mTOR/mTOR ratio compared to control cells (*p* = 0.0005). This effect was completely reversed by co-treatment with TAL (*p* = 0.0046 vs. oAβ_1−42_ group), which brought the ratio back to control levels (*p* = 0.5265; Fig. 3a, b).

Next, we assessed the activation state of AMPK, a known upstream regulator of mTOR. Our analysis of the p-AMPK/AMPK ratio revealed no statistically significant differences among any of the treatment groups (F_(3,16)_ = 0.6886, *p* = 0.5721; Fig. 3c, d). This suggests that the observed effects on mTOR occur independently of changes in global AMPK activation in this model.

We then measured the levels of p62/*SQSTM1*, a cargo receptor that accumulates when autophagic flux is impaired. Consistent with mTOR upregulation, oAβ_1−42_ treatment caused a significant increase in p62 levels (*p* = 0.0456). Co-treatment with TAL successfully reversed this accumulation, significantly reducing p62 levels compared to the oAβ_1−42_ group (*p* = 0.0093; Fig. 3e, f).

Finally, we analyzed the LC3-II/I ratio, a key marker of autophagosome formation. Changes in the LC3-II/I ratio may reflect alterations in autophagosome formation and/or degradation, since LC3-II is also degraded during autophagosome–lysosome fusion. In our model, the reduction in the LC3-II/I ratio together with p62 accumulation suggests impaired autophagic flux. Consistent with this, while the overall one-way ANOVA did not reach statistical significance (F_(3,18)_ = 2.906, *p* = 0.0630), post-hoc analysis revealed a significant decrease in the LC3-II/I ratio in response to oAβ_1−42_ treatment compared to the control group (*p* = 0.0413; Fig. 3g, h). This suggests a defect in the early stages of the autophagic process. Co-treatment with TAL did not produce a statistically significant reversal of this effect, preventing us from concluding a direct rescue of autophagosome formation itself.

### TAL Treatment Modulated the Gene Expression of Autophagy-Related Markers

**Fig. 4:**
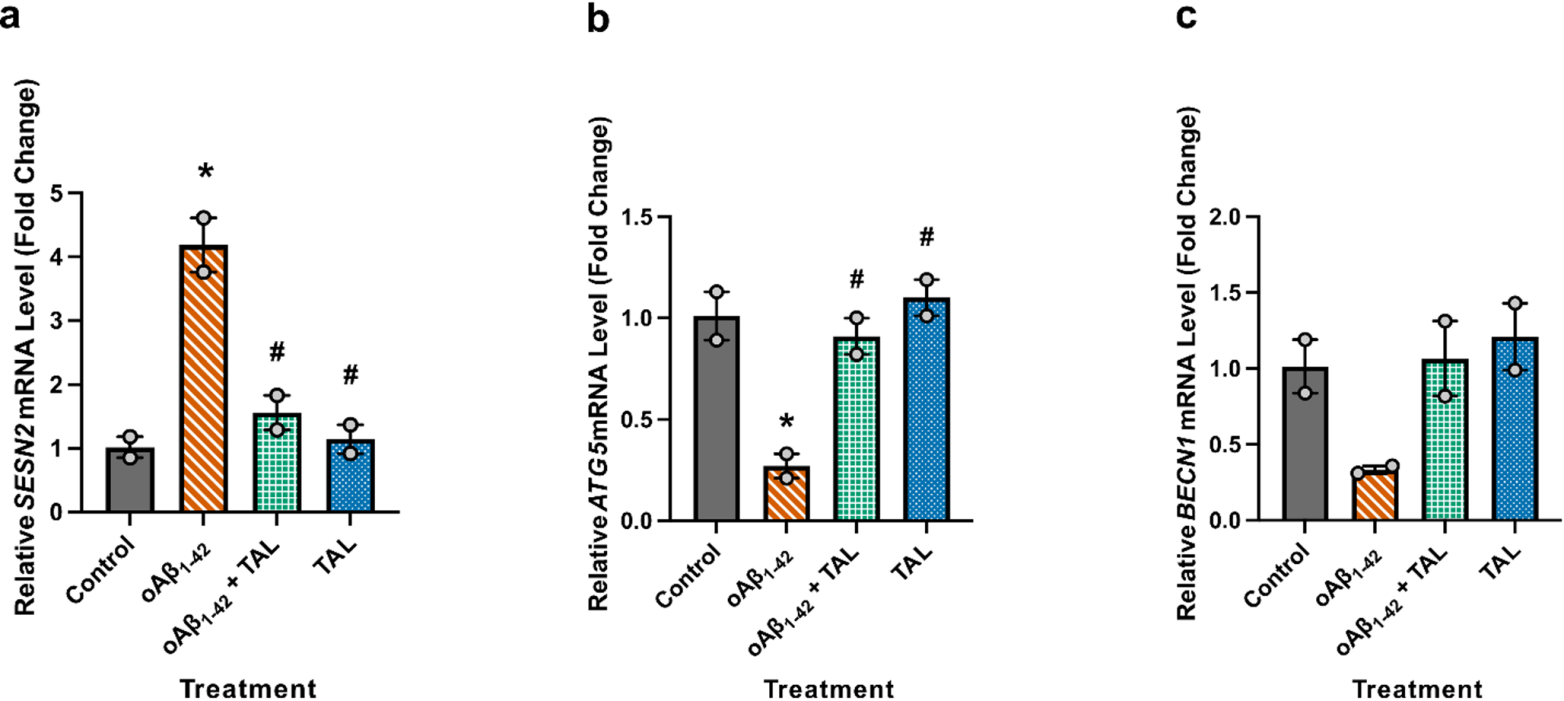
Effect of TAL on the gene expression of autophagy-related markers. HT-22 cells were treated for 32 h as indicated, and relative mRNA levels were assessed by RT-qPCR. Data are expressed as mean ± SEM (*n* = 2), normalized to ACTB, and shown as fold change relative to the control group, n indicates the number of independent biological experiments. Statistical analysis was performed using one-way ANOVA with Holm-Šídák’s multiple comparisons test. **a** oAβ_1−42_ significantly increased *SESN2* expression (*), an effect reversed by TAL (#). **b** oAβ significantly decreased *ATG5* expression (*), which was reversed by TAL (#). **c** No significant changes were observed for *BECN1* expression. *p < 0.05. * denotes a significant difference from control; # denotes a significant difference from the oAβ_1−42_ group

To further investigate the mechanisms underlying TAL’s effects, we examined the gene expression of several key autophagy-related markers using RT-PCR (Fig. 4). In cells exposed to oAβ_1−42_, a statistically significant increase in the expression of the stress-response gene *SESN2* was observed (*p* = 0.0088), an effect that was reversed by TAL co-treatment (*p* = 0.0119). A significant decrease in the expression of the autophagy initiation gene *ATG5* was also noted (*p* = 0.0238), which was similarly normalized by TAL (*p* = 0.0319). While a trend towards decreased expression was seen for *BECN1*, this did not reach statistical significance (F(3,4) = 4.35, *p* = 0.0948). These results demonstrate that TAL modulates the transcriptional regulation of key autophagy-related genes in response to oAβ_1−42_-induced stress.

### Total Endogenous GCase Levels are Unaffected by oAβ_1−42_ or TAL Treatment

**Fig. 5:**
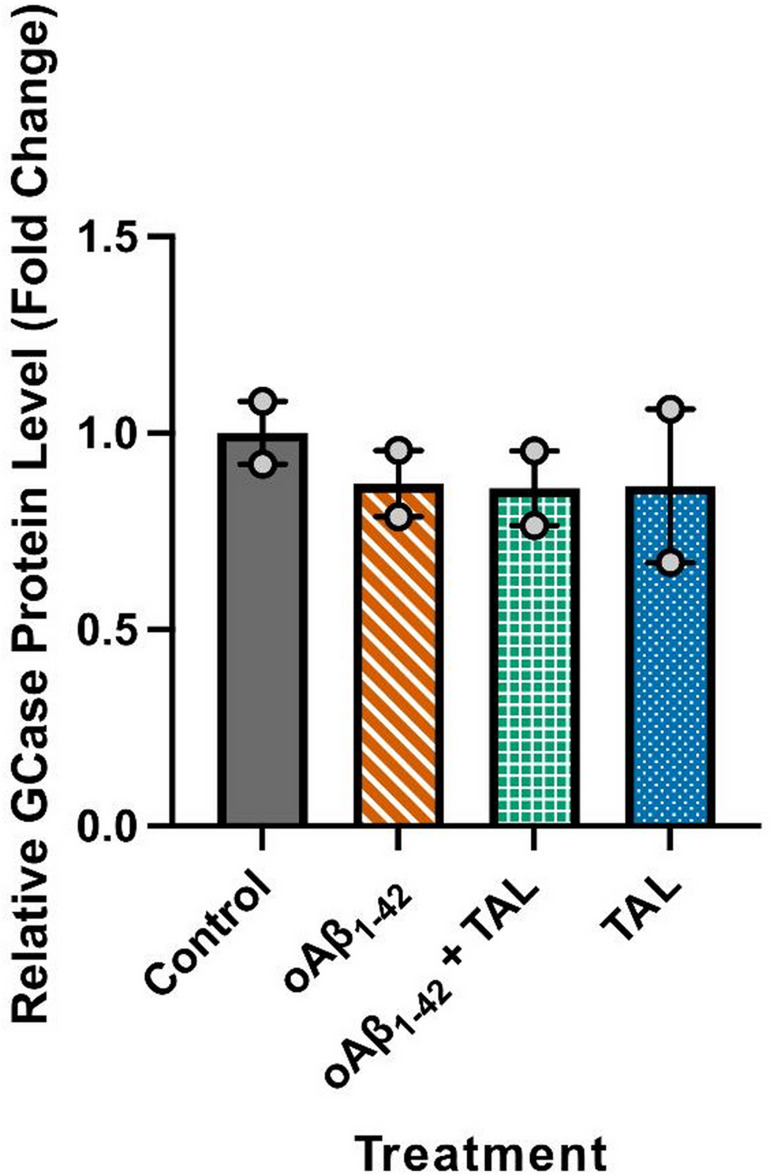
Total endogenous GCase levels are unchanged by oAβ₁₋₄₂ or TAL treatment. The relative amount of endogenous GCaseprotein in HT-22 cell extracts was measured by ELISA after 32 hours of treatment. Data are expressed as mean ± SEM (n=2)and shown as fold change relative to the control group, n indicates the number of independent biological experiments.Statistical analysis by one-way ANOVA revealed no significant differences among the groups

We next sought to determine if oAβ_1−42_ or TAL treatment affected the total levels of endogenous GCase protein. Using an ELISA on total cell extracts, we found that neither oAβ_1−42_ nor TAL treatment significantly altered the amount of endogenous GCase protein compared to the control (F(3,4) = 0.3011, *p* = 0.8242; Fig. 5). Consistent with Western blotting of total RIPA lysates, ELISA analysis of whole-cell PBS extracts did not demonstrate a significant change in total GCase protein levels following TAL exposure, indicating that TAL treatment does not alter bulk cellular GCase abundance. Additionally, to explore pathways related to GCase function, we measured the phosphorylation of LRRK2 but observed no significant changes in the pLRRK2/LRRK2 ratio in response to any treatment (Online Resource 1 Fig. S3).

## Discussion

The current findings suggest that TAL, a recombinant form of GCase, is associated with changes in intracellular Aβ levels and modulation of autophagy-related markers in HT-22 cells. These observations may indicate a potential interaction between TAL treatment and pathways involved in Aβ handling, although the underlying mechanisms remain to be clarified.

The amyloid hypothesis of AD, which asserts that Aβ plays a central role as the key peptide, has been widely accepted since 1992 [33, 34]. Aβ forms soluble, low molecular weight (LMW) oligomers in tetrameric, trimeric, and dimeric structures [35], which are considered toxic forms of the peptide before they deposit into amyloid plaques [36]. These oligomeric species are internalized and accumulate within neuronal cells [2–4]. It was suggested that release of Aβ_1−42_ monomers or oligomers into the cytoplasm and subsequent aggregation on microtubules may also be critical determinants of neurotoxicity [37, 38]. In this study, HT-22 cells treated with TAL at 8252 ng/mL alongside 5 µM oAβ_1−42_ for 32 h showed a reduction in monomeric Aβ signal (Fig. 2a, b), consistent with attenuation of oAβ_1−42_-associated burden under these conditions. Additionally, TAL treatment exerted a protective effect against the reduction in cell viability induced by 5 µM oAβ1–42 for 32 h, resulting in near-control viability levels (Fig. 1b). Because the MTT assay reflects mitochondrial metabolic activity rather than cell survival per se, TAL may mitigate metabolic stress rather than fully preventing Aβ-induced cell loss. We may speculate that the lack of a protective effect of TAL against Aβ-induced cytotoxicity at 24 h reflects insufficient accumulation within lysosomes, whereas the absence of protection at 48 h may be attributable to reduced or inadequate enzymatic activity relative to the 32-hour time point. In the prevalent model of AD, oligomers of Aβ are considered to be the main neurotoxic species; however, in the context of our studies, the monomeric forms of oAβ_1−42_, which are prone to conversion into neurotoxic oligomers, might also contribute to the progression of AD by exerting a partially modulatory effect on autophagy pathways.

The observed reduction in monomeric Aβ following TAL treatment may reflect a multifaceted influence on Aβ metabolism, potentially extending beyond degradation alone. By enhancing lysosomal GCase activity, TAL may improve lysosomal function and influence the local lipid environment [11, 39]. Improved lysosomal efficiency is thought to enhance degradation of Aβ precursors such as amyloid precursor protein (APP) and its C-terminal fragments, offering a possible route to reduced Aβ production [40]. Furthermore, altered membrane lipid composition, influenced by GCase activity, could modulate secretase activity and thereby contribute to Aβ regulation [41, 42]. Simultaneously, enhanced lysosomal activity may facilitate more efficient clearance of internalized soluble Aβ monomers [43]. This efficient removal is critical because endo-lysosomal compartments are key sites for intracellular Aβ accumulation and seeding [44, 45]. By reducing monomer concentration and preventing their accumulation within these vesicles, TAL directly disrupts the earliest stages of Aβ aggregation. This mechanism is key to inhibiting the formation of toxic oligomeric seeds before significant oligomerization can occur [45, 46]. Thus, TAL’s impact on lysosomal health and lipid homeostasis likely contributes by reducing the available Aβ monomer pool and by preventing the initial steps of aggregation.

Autophagy and lysosomal function are disrupted in individuals with AD and other neurodegenerative disorders linked to protein aggregation. Autophagic markers, such as Atg5, beclin-1, Atg12, and LC3, are also present in amyloid plaques and neurofibrillary tangles in the brains of AD patients [47, 48]. Furthermore, studies have demonstrated that APP and Aβ peptides co-localize with LC3-positive autophagosomes in neuroblastoma cells overexpressing APP and in AD mouse models [49, 50], suggesting that Aβ may be a substrate for autophagic degradation. Additionally, Aβ has been shown to enhance mTOR signaling, and a reduction in mTOR activity correlates with decreased Aβ levels, indicating a relationship between mTOR signaling and Aβ [51, 52]. Due to its hydrophobic carboxyl terminus [53], Aβ may disrupt intracellular organelle trafficking as well as the trafficking of autophagosomes and their fusion with lysosomes [54–56]. Furthermore, Aβ accumulation within lysosomes [57, 58] has been linked to impaired autophagy and lysosomal degradation [23, 59]. GCase deficiency and the loss of enzymatic activity have been associated with the pathological progression of AD and increased cell-to-cell spread of Aβ [11]. Limited information is available regarding the potential impact of GCase deficiency on AD pathology. Given the critical role of Aβ accumulation and lysosomal functionality in AD progression, it is hypothesized that overexpression of GCase could have significant effects in preventing disease progression. Studies have shown a significant reduction in GCase protein levels and enzymatic activity in postmortem hippocampal brain tissue of AD patients and primary neurons exposed to Aβ_1−42_ oligomers. Ectopic expression of GCase via lentivirus has corrected the impaired lysosomal activity and accelerated the degradation of Aβ_1−42_ oligomers, thereby protecting neurons from Aβ_1−42_-induced toxicity. Notably, the neuronal death caused by Aβ_1−42_ oligomers was found to correlate with a decrease in GCase protein levels and enzymatic activity, along with accompanying lysosomal biogenesis and acidification damage [11]. The increased Aβ aggregation and APP observed in GD mice further highlight the relationship between GCase dysfunction and AD [60]. In addition to the synaptic dysfunction observed in nerve cell death, it is suggested that Aβ_1−42_ causes lysosomal membrane permeabilization (LMP) due to a loss of lysosomal acidification and disruption of membrane integrity [11]. In primary neurons exposed to oAβ_1−42_, an increase in intracellular acidification, along with a decrease in lysosome number and size, suggests that LMP plays a role in Aβ toxicity [11]. GCase expression, however has been shown to reverse Aβ oligomer-induced LMP [11]. In AD patients, the expression and enzymatic activity of GCase are reduced, contributing to lysosomal dysfunction and playing a significant role in the development of AD [11]. Gene therapy targeting the *GBA1* gene that encodes GCase accelerates Aβ oligomer clearance through increased lysosomal function and protects neurons from Aβ_1−42_-induced cell death [11]. Pharmacological ERT aimed at restoring GCase or enhancing lysosomal function could be a potential new therapeutic strategy to prevent the progression of AD pathology.

Our observation that TAL is associated with an increased GCase signal in lysate fractions enriched for lysosomal proteins may be indicative of lysosomal association however, direct confirmation of compartment-specific delivery was not performed in the present study. TAL uptake is generally thought to occur via receptor-mediated endocytosis, a mechanism fundamentally different from the intracellular trafficking of the native enzyme [9]. Neurons are known to express at least two relevant carbohydrate-recognizing receptor systems, including the mannose receptor (MR) and the mannose-6-phosphate receptor (M6PR). TAL is specifically glycoengineered with terminal mannose residues which may facilitate interaction with MR; a pathway known to mediate the clearance of glycoproteins and even Aβ itself, however, the specific uptake pathway was not directly examined in this study [61]. This is its primary intended route of entry, leveraging a natural feature of its plant-based production [9]. Therefore, the proposed mechanism of cellular entry should be considered as putative and requires further experimental validation.

The topic of M6P-dependent trafficking in relation to GCase has been a subject of recent scientific debate. This stems from a structural study suggesting that the GCase escort protein, LIMP-2, could itself be M6P-tagged and targeted by the cation independent CI-M6PR [62]. However, our manuscript’s citation of Blanz et al. [10] aligns with the current consensus, which is based on compelling *in vivo* evidence showing that the physiological, intracellular sorting of native GCase via LIMP-2 is definitively M6P-independent.

This distinction is critical: the LIMP-2 pathway is for the internal sorting of the cell’s own GCase [9, 10], whereas TAL is an external agent requiring entry through the cell membrane. While the M6P pathway is not TAL’s primary target, the work by Mathews et al. (2002) [63] demonstrates that M6PR overexpression can lead to the mislocation of the active form of the key lysosomal protease cathepsin D and exacerbate Aβ pathology. Cathepsin D has been shown to utilize the type 1 transmembrane protein SEZ6L2 as a specific, M6P-independent transport receptor to reach endolysosomes in neuronal cells This reliance on M6P-independent sorting is emerging as a crucial feature of neuronal lysosomal biology, as neurons appear less dependent on the canonical M6P pathway than other cell types [64]. The existence of such specialized pathways highlights the general vulnerability of lysosomal trafficking systems in AD and reinforces the importance of a direct and efficient delivery route, such as the one provided by TAL’s engagement with the MR. It has also been shown that inactivation or mislocalization of cathepsin D leads to neuronal ceroid lipofuscinosis (NCL), an orphan neurodegenerative disorder [65–67]. In our previous study, we demonstrated *in vitro* the effect of a TPP1 analogue, previously used in NCL, in AD [68]. These and similar studies suggest that drugs developed for lysosomal storage disorders may have potential applications in other neurodegenerative diseases.

Our findings suggest that taliglucerase alfa, ERT, is associated with changes in intracellular Aβ levels and modulation of autophagy-related markers in a neuronal model, consistent with a growing body of evidence supporting a potential role of lysosomal enzyme augmentation in neurodegenerative diseases.

In this study, the autophagy-related proteins p-AMPK/AMPK, p62/*SQSTM1* and LC3-II/I were measured in HT-22 cells, along with the assessment of mTOR, the central regulator of autophagy (Fig. 3), which coordinates autophagic activity through sequential actions of Atgs [69]. Although LC3-II abundance is commonly used as a marker of autophagosome formation, it also depends on its lysosomal turover rate. Therefore, LC3-II levels should ideally be interpreted in the context of autophagy flux assays. In this study, LC3-II changes were evaluated together with p62 levels to better infer the functional status of the autophagy pathway [70]. Lysosomal fusion and protease activity are critical for autophagy, and any abnormalities in this phase can impair cargo degradation, even if other steps in the autophagic pathway function normally. p62/*SQSTM1* is an autophagosome cargo protein involved in protein turnover by initiating lysosomal degradation via autophagic flux. The LC3-interacting region of p62/*SQSTM1* promotes selective autophagy by interacting with LC3 [20, 71, 72]. Treatment of HT-22 cells with oAβ_1−42_ resulted in a significant increase in the p-mTOR/mTOR ratio (Fig. 3a, b) and p62/*SQSTM1* levels (Fig. 3e, f), which was critically accompanied by a significant decrease in the LC3-II/I ratio (Fig. 3g, h). In addition, treatment with TAL, either alone or in combination with oAβ_1−42_, promoted the conversion of the precursor form of cathepsin B (pro-catB; 43–45 kDa), observed in the lysosome-enriched extracts of control and oAβ_1−42_-treated cells, into the fully active (enzymatically active; mature; 25–27 kDa) double-chain form consisting of heavy and light subunits (Fig. 2c). This finding is particularly significant as the processing of pro-enzymes into their active forms is a hallmark of a healthy and functional lysosomal environment. The presence of mature cathepsin B suggests that TAL treatment may support lysosomal proteolytic competence beyond GCase delivery, which is important for the efficient degradation of autophagic cargo and depends on the coordinated activity of multiple lysosomal hydrolases. The proper trafficking and activation of lysosomal proteases, such as Cathepsin D as explored by Boonen et al. [64], is fundamental to neuronal homeostasis, and our data suggest that enhancing GCase function bolsters this entire system.

Although TAL alone did not induce significant changes in p-mTOR/mTOR or p62, the combined treatment of TAL and oAβ_1−42_ significantly reversed the oAβ_1−42_-induced elevation of both p-mTOR/mTOR and p62 levels (Fig. 3b, f).The reduction of p62, in particular, supports an interpretation of enhanced autophagy-related clearance, while flux per se will benefit from direct, dynamic readouts. Although the trend toward a reduced LC3-II/I ratio was not significantly reversed by TAL, this may reflect the dynamic nature of autophagy, in which autophagosomes can be cleared at rates comparable to their formation. In parallel, modulation of *ATG5* and *BECN1* expression (Fig. 4b, c), including a significant increase in *ATG5* levels, is consistent with a supportive effect of TAL on autophagy-related machinery. The trend towards normalization of *BECN1* gene expression by TAL (Fig. 4c) may be particularly significant, as Beclin 1 is not only a key initiator of autophagy but has been shown to be fundamentally required for neuron viability through its crucial roles in the endosomal pathway [73]. Interpreting these findings together reveals a complex, multi-stage disruption of the autophagy pathway by oAβ_1−42_. The significant decrease in the LC3-II/I ratio points to an impairment in the early stage of autophagy, specifically a reduction in autophagosome formation. Simultaneously, the profound accumulation of the cargo protein p62 indicates a failure in the later stages, namely the clearance and degradation of autophagic substrates. This dual-impact phenotype—reduced formation and impaired clearance—suggests a comprehensive shutdown of functional autophagic flux.

The induction of sestrin-dependent AMPK activation and the suppression of mTORC1 activity are critical for the maintenance of basal autophagy [74]. Sestrin-mediated inhibition of mTOR is also essential for the autophagic degradation of proteins that inhibit antioxidant genes. Through AMPK activation, SESN2 can inhibit enzymes that produce pathogenic levels of reactive oxygen species (ROS) [75]. Low levels of oxidative stress stimulate sestrins, reducing oxidative stress and preventing cell death [27, 76]. In this way, sestrins function as genetic components involved in cell viability and function, eliminating the inevitable consequences of oxidative stress. Sestrin-mediated mTOR inhibition also plays a key role in the autophagy-dependent degradation of proteins that suppress antioxidant gene expression. In the ischemic-damaged mouse brain, increased expression of SESN2 has been demonstrated [76, 77]. The antioxidant and, particularly, the autophagy-inducing effects of sestrins have increased their relevance in neurodegenerative diseases. Exposure of CHP-134 neuroblastoma cells to Aβ_1−42_ increased SESN2 expression [78, 79]. In primary rat cortical neuronal cultures, Aβ was observed to cause an increase in SESN2, activating antioxidant and autophagy pathways. In the widely used transgenic AD animal model, the 12-month-old APPswe/PSEN1dE9 mice, an increase in SESN2 expression was observed in the cortex. A concurrent increase in the autophagosome marker LC3-II was also observed in the same cell culture and animal model. The increase in SESN2 caused by Aβ was reversed by *SESN2* siRNA, and a decrease in LC3-II accompanied this reversal. Additionally, knockdown of *SESN2* and pharmacological inhibition of autophagy with bafilomycin A enhanced neuronal damage induced by Aβ. These findings suggest that SESN2 induction or inhibition is closely linked to AD, and autophagy pathways play a crucial role in this relationship [80]. In our study, treatment of HT-22 cells with oAβ_1−42_ for 32 h led to a significant increase in *SESN2* gene expression (Fig. 4a) and a significant decrease in *ATG5* expression (Fig. 4b). These genetic changes occurred alongside markers of autophagy dysfunction: elevated p-mTOR/mTOR (Fig. 3b) and p62 levels (Fig. 3f), and a significant decrease in the LC3-II/I ratio (Fig. 3h), which indicates impaired autophagosome formation. TAL treatment normalized *SESN2* and *ATG5* gene expression toward control levels (Fig. 4a, b) and was associated with indicators of improved autophagic flux. These findings demonstrate that although oAβ_1−42_ induces a compensatory upregulation of *SESN2*, this response is insufficient to overcome the concurrent suppression of *ATG5* and activate the autophagy pathway. Moreover, despite the Aβ-induced increase in *SESN2*, there was no statistically significant elevation in the p-AMPK/AMPK ratio (Fig. 3d), indicating that Aβ suppresses autophagic pathways independently of *SESN2*-mediated compensation. In contrast, TAL appears to act by modulating the expression of key autophagy-related genes, which may contribute to attenuation of the cellular stress response.

The normalization of *SESN2* levels by TAL, together with markers consistent with autophagy pathway improvement in the absence of global AMPK activation, can be understood through several AMPK-independent mechanisms. Firstly, *SESN2* itself can directly suppress mTORC1 by interacting with the GATOR complex on lysosomes and can also associate with the ULK1 initiation complex and p62/*SQSTM1* to facilitate autophagic clearance [81]. Secondly, TAL’s primary action of restoring GCase leads to improved lysosomal function, such as enhanced substrate degradation [82], which in turn enhances autophagic flux [83], helps normalize lysosomal pH [84], and reduces aberrant mTORC1 signaling originating from dysfunctional lysosomes [82]. By alleviating Aβ-induced cellular stress, including proteotoxic and oxidative components, and supporting lysosomal clearance [11], TAL may reduce upstream stimuli associated with SESN2 overexpression [85]. Consequently, with the upstream stimuli reduced, the cell no longer requires a heightened SESN2 stress response, leading to its normalization [81]. Furthermore, the restored lysosomal environment allows even basal levels of SESN2 to more efficiently regulate mTORC1 [81] and support the now more effective autophagic machinery [82].

### Limitations of the Study

The present study provides valuable insights into the potential neuroprotective mechanisms of TAL in an *in vitro* model of Aβ toxicity. However, several limitations should be acknowledged. Firstly, all experiments were conducted using the immortalized mouse hippocampal cell line HT-22. While a well-established model for initial mechanistic studies, findings may not fully recapitulate the complex pathology of the human brain in AD, underscoring the need for future validation in primary neuronal cultures or *in vivo* animal models.

Secondly, our study did not directly measure the catalytic activity of GCase, lipidomic analyses, and lipid homeostasis following TAL treatment or formally assess autophagic flux using inhibitors like bafilomycin A1. While the observed accumulation of GCase in lysosomes and the clearance of p62 strongly support our conclusions, direct functional assays would provide definitive confirmation in future work.

In addition, complementary analyses of other lysosomal membrane proteins, such as LAMP-1, as well as lysosomal proteases, including cathepsin D or cathepsin L, would have further strengthened the interpretation that TAL modulates lysosomal function.

Furthermore, while our data point to enhanced intracellular clearance, we did not investigate potential effects of TAL on Aβ uptake or its extracellular aggregation state. Further investigations are also required regarding the entry of TAL into neuronal cells, particularly into lysosomes.

In addition, some molecular analyses were conducted with a limited number of independent biological replicates, and future studies with larger sample sizes will be required to confirm these findings.

Accordingly, the lack of complementary morphological analyses, including immunocytochemistry, constitutes a limitation of the present study and should be addressed in future investigations. GBA was detected in lysosome-enriched extracts, which are not organelle-pure preparations and inherently yield lower protein amounts compared to whole-cell lysates; therefore, further studies are needed to elucidate the accumulation and localization of TAL within lysosomes.

Finally, a critical hurdle for the clinical translation of these promising *in vitro* findings is the limited ability of large biologic molecules like TAL (MW ~ 61 kDa) to cross the blood-brain barrier (BBB). Prior studies have shown poor CNS penetration of similar ERTs. Therefore, any future *in vivo* testing will necessitate the development of brain-targeted delivery systems, such as fusion with BBB-shuttles or nanoparticle encapsulation, to be therapeutically viable for AD.

**Fig. 6:**
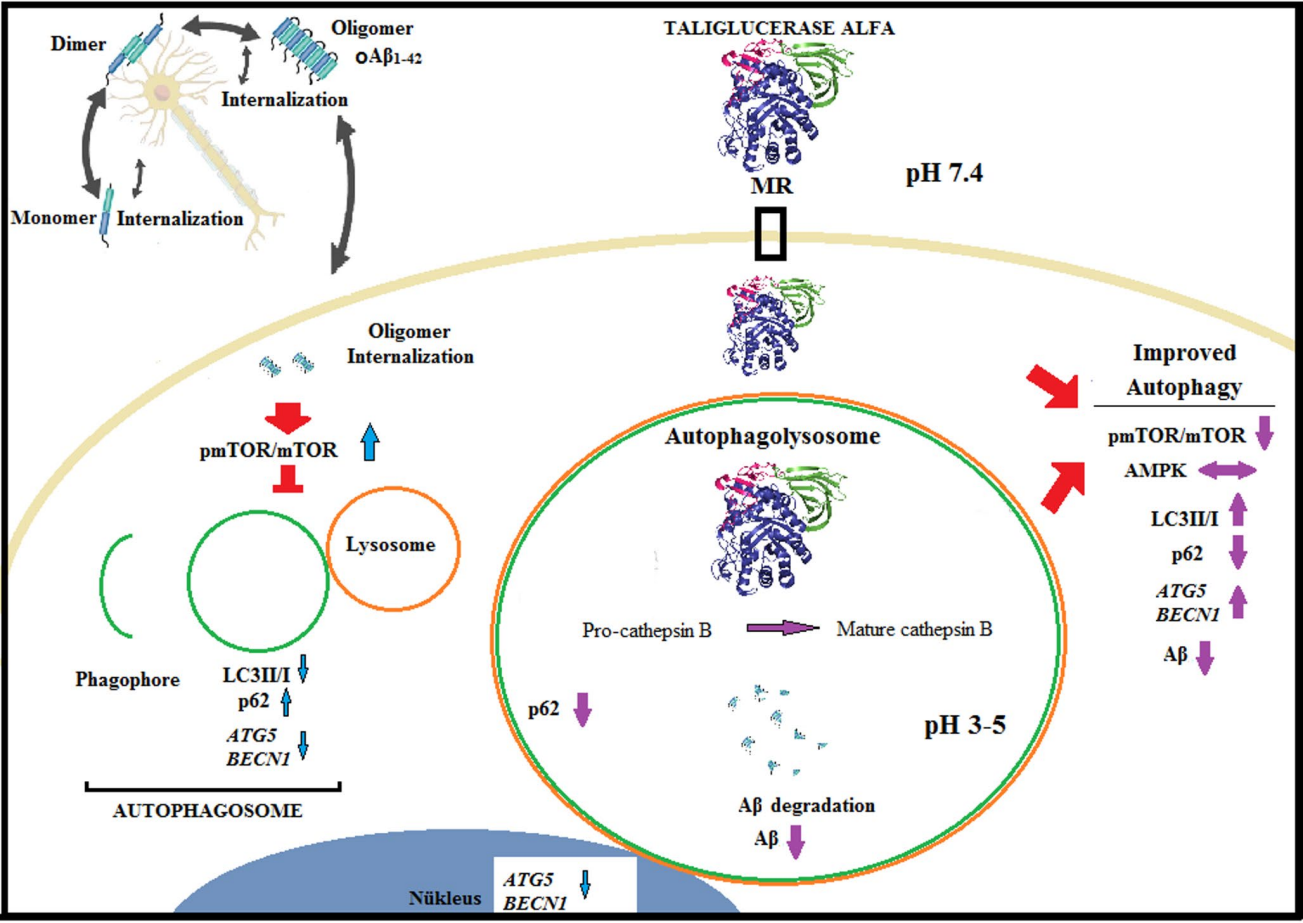
Schematic representation of the proposed mechanistic actions of taliglucerase alfa (TAL)

## Conclusion

In this exploratory, proof-of-concept study, TAL treatment was associated with modulation of autophagy-related markers and a reduction in intracellular monomeric Aβ levels in an *in vitro* neuronal model of AD. These observations may be linked to lysosomal GCase accumulation and changes in lysosomal proteolytic components, including cathepsin B maturation; however, the underlying mechanisms were not directly established. The observed changes in mTOR signaling, p62 levels, and selected autophagy-related proteins suggest a potential interaction between TAL treatment and autophagy-associated processes, although functional autophagic flux was not directly assessed. Taken together, these findings provide preliminary, hypothesis-generating evidence supporting a potential relationship between lysosomal GCase augmentation and Aβ- and autophagy-related pathways in Alzheimer’s disease. Beyond protein clearance, lysosomal function may also contribute to broader aspects of neuronal homeostasis; however, these possibilities remain to be clarified.

Furthermore, it is worth considering that the benefits of improving lysosomal health may extend beyond the clearance of pathogenic aggregates. The lysosome is increasingly recognized as a key signaling hub, and lysosomal enzymes can have specific, regulated functions, such as the modulation of neurite outgrowth by Cathepsin D through cleavage of its receptor SEZ6L2 [64]. An intriguing avenue for future research would be to explore whether restoring GCase function with TAL confers broader benefits to neuronal structure and homeostasis, in addition to its effects on proteostasis.

Further studies incorporating expanded datasets, direct functional assessments, and *in viv*o models notably the BBB penetration of TAL will be required to validate these findings and to better define the mechanistic and translational relevance of lysosomal GCase modulation in AD.

## Materials and Methods

### Cell Culture and Treatment

Mouse hippocampal neurons (HT-22; passage number 9) were donated by Atlas Biotechnology (Ankara, Turkey). The cells were grown in Dulbecco’s modified Eagle’s medium (DMEM) (Cegrogen Biotech, Stadtallendorf, Germany, cat. #E0500-160) supplemented with 10% fetal bovine serum (FBS) (Cegrogen Biotech, Stadtallendorf, Germany, cat. #A0500-3210), 1% L-glutamine (200 mM) (Cegrogen Biotech, Stadtallendorf, Germany, cat. #K0100-670) and 1% penicillin/streptomycin (10,000 U/mL) (Cegrogen Biotech, Stadtallendorf, Germany, cat. #P0100-790) at 37 °C in an incubator with a humidified CO2 environment of 5%.

TAL, a recombinant analog of GCase, is a licensed and patented product of Pfizer [86], and donated by the company for this study.

Cell viability experiments were performed using TAL (ELELYSO^®^, Pfizer, USA) 1656 and 8252 ng/mL. TAL was administered when cell confluency reached 50% in 96-well plates. For the treatment of HT-22 cells, TAL 8252 ng/mL final concentration was used. Control cells were exposed to the same quantity of artificial cerebrospinal fluid (aCSF). TAL was administered when cell confluency reached 70%-80% in 6-well plates. The cells were exposed to 5 µM oAβ_1−42_ and concomitant TAL incubation for 32 h. Following incubation, the treatment media was removed and the cells were washed 3 times with phosphate-buffered saline (PBS) with pH 7.4 at 37 °C to remove residual oAβ_1−42_. For whole cell extract, cells were then lysed with radioimmunoprecipitation assay (RIPA) buffer (Tris-HCl 50 mM [pH 7.4], NaCl 150 mM, NP-40 1%, sodium deoxycholate 0.5%, SDS 0.1% [Boston Bioproducts, Worchester WA, USA, cat. #BP-115]) supplemented with protease (Complete Protease Inhibitor Cocktail, Roche, Basel, Switzerland, cat. # 11697498001) and phosphatase inhibitors (dithiothreitol DTT, Amresco In ., Solon, OH, USA, cat. #97061-340), and centrifuged at 14 000 g for 20 min at 4 ° C in a microcentrifuge. The protein concentration was determined via Bradford assay. The extracts were stored at -80 °C until western blotting.

### Preparation of oAβ_1−42_

Aβ_1−42_ human peptide (lyophilized, 1 mg) (Novex by Life Technologies, USA, lot #75555483A) was dissolved in sterile water (molecular biology grade) and diluted in Ca^2+^ free PBS at a concentration of 2 mM and incubated at 37 °C for 24 h. Aβ_1–42_ peptide was prepared following the protocol of Kasza et al., which yields preparations enriched in low–molecular weight oligomeric assemblies. All preparations were handled under identical conditions across experiments. However, the oligomeric state of the preparations used in this study was not independently confirmed by Western blot or other biophysical methods.The preparation was centrifuged at 14,000 g for 10 min at 4 °C, and the supernatant containing soluble oligomeric Aβ_1−42_ was transferred to clean tubes and stored at 4 °C. oAβ_1−42_ was used within 24 h after preparation [87].

### Cell Viability Assay

96-well plates were seeded with 1.5 × 10^4^ cells per well 1 day before treatment and allowed to adhere overnight to reach 50% confluency. This experiment was carried out using a stock solution of 3-[4,5-dimethylthiazol-2-yl]-2,5-diphenyltetrazolium bromide (MTT), which was diluted in dimethyl sulfoxide (50 mg/mL) as a 100-fold stock solution [88]. At the end of TAL and/or oAβ_1−42_ treatment, HT-22 cells were incubated in the culture medium with MTT (0.5 mg/mL) in the dark at 37 °C for 4 h to allow the living cells to form insoluble formazan precipitates. Following incubation, 150 µL of isopropyl alcohol was added to each well, and the plates were agitated for 5 min to solubilize the crystals. Absorbance at a wavelength of 570 nm was measured using a plate reader (Biotek Instruments Inc., Winooski, VT, USA).

### RT(Q)-PCR Studies

A total RNA isolation system (Nzytech, Lisboa, Portugal) was used to extract total RNA from HT-22 cells, and the purity of the recovered RNA was validated spectrophotometrically at 260/280 nm. Utilizing an RT-PCR kit, the RNA was reverse-transcribed and used to create complementary DNA (Strata Gene, La Jolla, CA, USA). According to the manufacturer’s recommended thermal cycling methodology, quantitative RT-PCR was performed using SYBR Green JumpStart Taq ReadyMix (Sigma-Aldrich, St. Louis, MO, USA, cat. # S9194-20RXN). β-actin (ACTB) was used as an internal control for mRNA expression. Results represent the fold change in the expression of target genes (relative to the control) calculated using the 2^−ΔΔCt^ method [89]. The following oligonucleotides were used: *SESN2*: fw 5’: 5-tag cctgcagcctcacct at-3, rev 5’: tatctgatgccaaagacgca; *ATG5*: fw: 5′-gcagatggacagttgcacacac-3′, rev: 5′- gaggtgtttccaacattggctca-3′; *BECN1*: fw: 5′-ctggacactcagctcaacgtca-3′, rev: 5′-ctctagtgccagctcctttagc − 3′; *ACTB*: fw: 5′-caccattggcaatgagcggttc-3′, rev: 5′- aggtctttgcggatgtccacgt-3′.

### Lysosome-Enriched Extracts Preparation and Western Blotting

For detergent soluble, lysosome-enriched extracts preparation, cells cultured in 6-well plates were washed with cold phosphate-buffered saline (PBS). The culture medium was collected and cleared by centrifugation (2,000 g, 10 min, 4 °C), and the supernatant was stored. The cells were lysed directly in the wells with 120 µL of ice-cold RIPA buffer (Boston BioProducts, Ashland, MA, USA; cat. #BP-115) supplemented with protease and phosphatase inhibitors and %0.1 Tween 20. The cell lysate was collected and subjected to two cycles of freeze-thaw and sonication (5 short bursts on ice) to ensure lysosomal membrane disruption. After a final incubation on ice for 30 min, the lysate was clarified by centrifugation at 20,630 g for 10 min at 4 °C. The supernatant, enriched with lysosomal contents, was collected for analysis.

Protein concentrations for both total cell lysates (RIPA) and lysosome-enriched extracts were determined using a BCA protein assay. Equal quantities of protein were separated on 4%-20% Mini-PROTEAN^®^ TGX™ Precast Protein Gels (Bio-Rad, Hercules, CA, USA; cat. #4561094) and transferred to Immun-Blot^®^ PVDF membranes (Bio-Rad; cat. #1620177). Membranes were blocked with 5% non-fat dry milk or bovine serum albumin (BSA) in Tris-buffered saline with 0.1% Tween-20 (TBS-T) for 1 h at room temperature. Membranes were then incubated overnight at 4 °C with the following primary antibodies: anti-β-Amyloid (D54D2) XP^®^ Rabbit mAb (1:1000; Cell Signaling Technology, Danvers, MA, USA; cat. #8243S), anti-mTOR (7C10) Rabbit mAb (1:1000; Cell Signaling Technology; cat. #2983S), anti-Phospho-mTOR (Ser2448) (D9C2) XP^®^ Rabbit mAb (1:1000; Cell Signaling Technology; cat. #5536S), anti-AMPKα (D63G4) Rabbit mAb (1:1000; Cell Signaling Technology; cat. #5832), anti-Phospho-AMPKα (Thr172) (D4D6D) Rabbit mAb (1:1000; Cell Signaling Technology; cat. #50081), anti-LRRK2 [MJFF2 (c41-2)] Rabbit mAb (1:1000; Abcam, Cambridge, UK; cat. #ab133474), anti-LRRK2 (phospho S935) [UDD2 10(12)] Rabbit mAb (1:1000; Abcam; cat. #ab133450), anti-GBA [2E2] (1:1000; Abcam; cat. #ab55080), anti-SQSTM1/p62 [2C11] (1:5000; Abcam; cat. #ab56416), anti-LC3B (1:5000; Abcam; cat. #ab48394), cathepsin B (H-5) Antibody (1:500; Santa Cruz Biotechnology, cat. #sc-365558), and anti-β-Actin (8H10D10) Mouse mAb (1:1000; Cell Signaling Technology; cat. #3700S).

After washing in TBS-T, membranes were incubated with the appropriate HRP-conjugated secondary antibodies: Anti-rabbit IgG, HRP-linked Antibody (1:2000; Cell Signaling Technology; cat. #7074S) or Anti-mouse IgG, HRP-linked Antibody (1:2000; Cell Signaling Technology; cat. #7076S). Protein bands were visualized using WesternBright™ Sirius™ HRP substrate (Advansta, San Jose, CA, USA; cat. #K-12043-D10) and imaged on a Kodak Image Station 4000 MM (Carestream, Rochester, NY, USA). β-actin served as the loading control for normalization. The BLUelf™ Prestained Protein Ladder (GeneDireX, Taoyuan City, Taiwan; cat. #PM008-0500) was used to approximate molecular weights.

### ELISA Assays

Total GCase protein levels were quantified using a commercial ELISA kit applied to PBS-based whole-cell extracts. The microplate provided in this kit (BT Lab, Wuhan, China; Mouse glucosidase beta; cat. #E0746Mo) has been pre-coated with an antibody specific to GCase. Standards or samples are then added to the appropriate microplate wells with a biotin-conjugated antibody specific to GCase. Next, Avidin conjugated to Horseradish Peroxidase (HRP) is added to each microplate well and incubated. After TMB substrate solution is added, only those wells that contain GCase, biotin-conjugated antibody and enzyme-conjugated Avidin will exhibit a change in color. The enzyme-substrate reaction is terminated by the addition of sulphuric acid solution and the color change is measured spectrophotometrically at a wavelength of 450 nm ± 10 nm. The concentration of Gcase in the samples is then determined by comparing the O.D. of the samples to the standard curve.

### Statistical Analysis

All statistical analyses were performed using GraphPad Prism version 10.3.1 (GraphPad Software, La Jolla, CA, USA). Data are presented as mean ± standard error of the mean (SEM) unless otherwise noted. The specific statistical tests used are detailed in the corresponding figure legends. In general, comparisons between multiple groups were made using one-way or two-way analysis of variance (ANOVA) as appropriate for the experimental design. Post-hoc tests were selected to appropriately test the specific hypotheses, and included Tukey’s HSD for all-pairs comparisons, Dunnett’s test for comparisons against a single control, and the Holm-Šídák test for pre-planned comparisons. For all analyses, a p-value < 0.05 was considered statistically significant. For all quantitative analyses, n refers to the number of independent biological experiments performed on different days using separately prepared cell cultures. Each biological replicate included 3 technical wells per condition, and the mean value of the technical replicates was used for statistical analysis.

## Supplementary Information

Below is the link to the electronic supplementary material.

Supplementary Material 1

Supplementary Material 2

## Data Availability

The raw data supporting the conclusions of this article will be made available by the authors upon reasonable request.
