# Supplementary material for: Taliglucerase Alfa Reduces Amyloid-β Burden by Restoring Autophagic Pathways in a Neuronal Model of Alzheimer’s Disease
Source: Neurochem Res. 2026 Jun 1;51(3):181. doi: 10.1007/s11064-026-04792-w (PMC13226380; doi:10.1007/s11064-026-04792-w)
Supplement: Supplementary file 1 — Supplementary Material 1 [file 11064_2026_4792_MOESM1_ESM.pdf]

Taliglucerase Alfa Reduces Amyloid-β Burden by Restoring Autophagic Pathways in a Neuronal Model of Alzheimer's Disease

Çağrı Özkurt[1] (ORCID: 0000-0001-9641-6745), Selma Köse[2] (ORCID: 0000-0001-6682-3569), Çimen Karasu[3] (ORCID: 0000-0002-0954-8465), Arjan Kortholt[4,5] (ORCID: 0000-0001-8174-6397), Pelin Kelicen-Uğur[1*] (ORCID: 0000-0003-1661-0631)

[1] Hacettepe University, Faculty of Pharmacy, Department of Pharmacology, Ankara, Turkey

[2] Lokman Hekim University, Faculty of Pharmacy, Department of Pharmacology, Ankara, Turkey

[3] Gazi University, Faculty of Medicine, Department of Medical Pharmacology, Ankara, Turkey

[4] Suleyman Demirel University, YETEM-Innovative Technologies Application and Research Centre, Isparta, Turkey

[5] Department of Cell Biochemistry, University of Groningen, Groningen, The Netherlands

*Correspondence: Pelin Kelicen-Uğur pkelicen@hacettepe.edu.tr

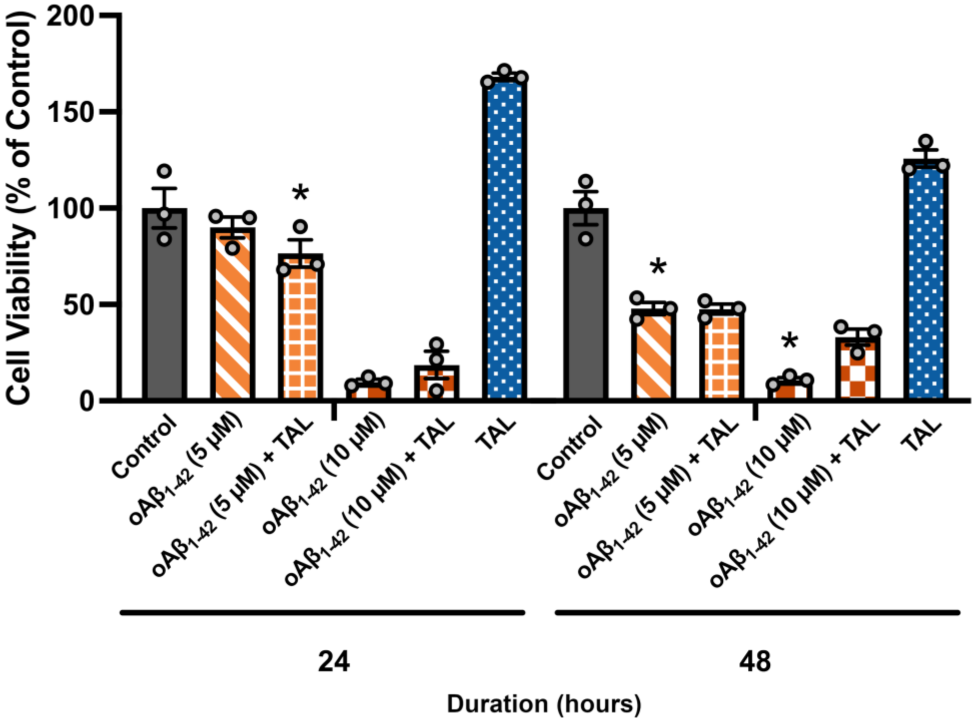

Fig. S1 Time-course analysis of oAβ_{1-42}-induced cytotoxicity and the neuroprotective effect of TAL at 24 and 48 hours. HT-22 cell viability was assessed by MTT assay following exposure to 5 µM or 10 µM oAβ_{1-42}, with or without co-treatment with 8252 ng/mL TAL. Data are presented as mean ± SEM (n=3-5). At 24 hours, only the 10 µM concentration of oAβ_{1-42} induced significant cytotoxicity, which was not rescued by TAL. At 48 hours, both concentrations of oAβ_{1-42} were cytotoxic; TAL showed only a non-significant trend toward rescuing viability in the 10 µM oAβ_{1-42} group ($p=0.0589$). These results informed the selection of the 32-hour time point for the main experiments (Fig. 1), as it provided the optimal model for observing both significant toxicity and a robust protective effect. Statistical analysis was performed using one-way ANOVAs at each time point, followed by Tukey's multiple comparisons test. Significance is denoted as $p < 0.05$. * indicates a significant reduction in viability in the oAβ_{1-42} group compared to the time-matched Control group.

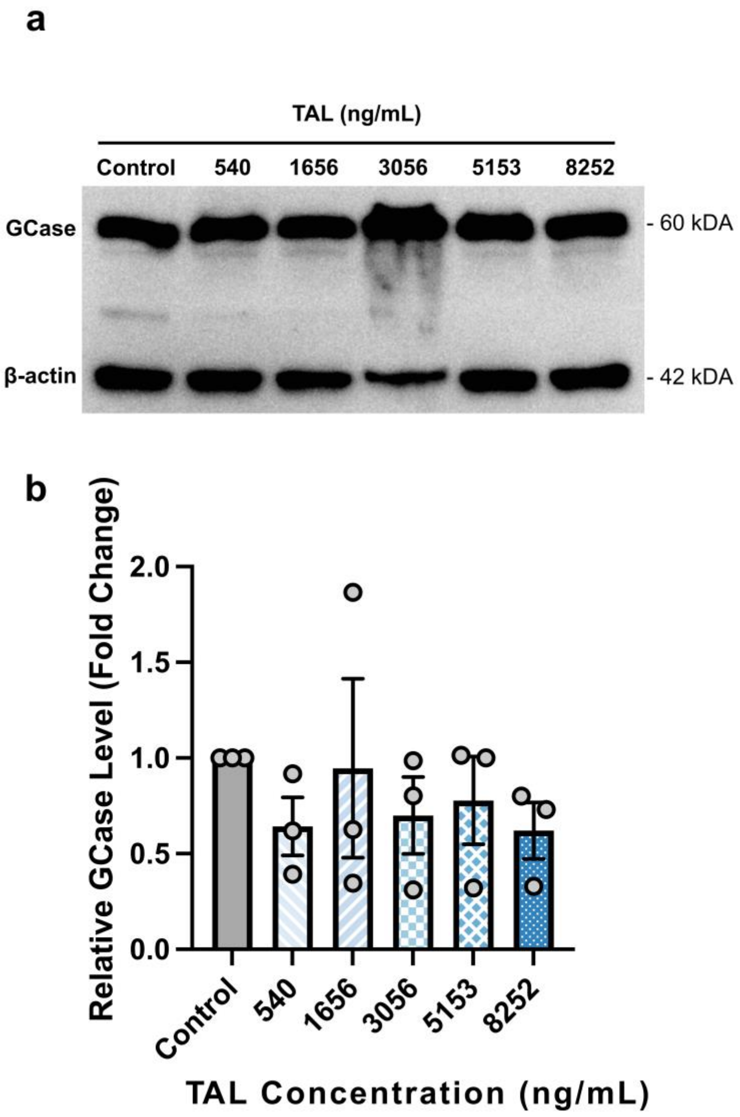

Fig. S2 Increasing concentrations of Taliglucerase alfa (TAL) do not alter total GCase protein levels in HT-22 cells. HT-22 cells were treated for 32 hours with the indicated concentrations of TAL. (a) Representative Western blot showing GCase (~60 kDa) and the loading control β-actin (~42 kDa) in total cell lysates. (b) Densitometric analysis of GCase levels, normalized to β-actin. Data are expressed as fold change relative to the control group and are presented as mean ± SEM (n=3). A one-way ANOVA confirmed no significant differences among the treatment groups ($F_{(5,12)} = 0.4272$, $p = 0.8212$)

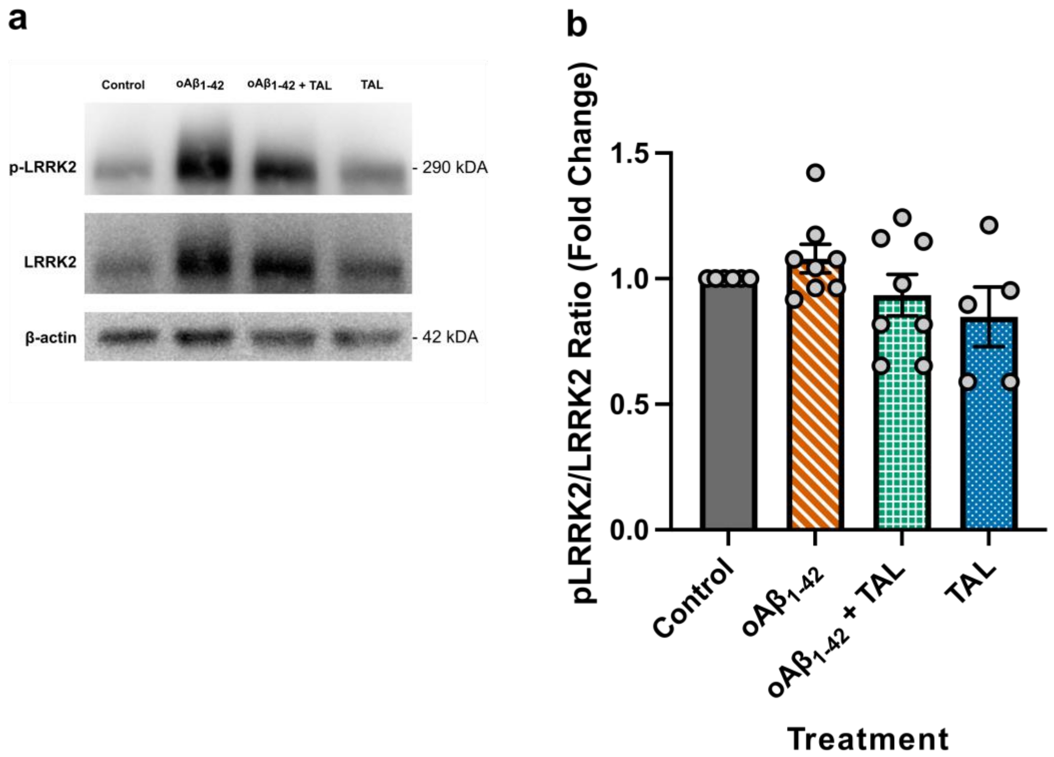

Fig. S3 LRRK2 phosphorylation is not altered by oAβ_{1-42} or TAL treatment. HT-22 cells were treated for 32 hours as indicated. (A) Representative Western blot for phosphorylated LRRK2 (pLRRK2, Ser935) and total LRRK2. (B) Densitometric analysis of the pLRRK2/LRRK2 ratio, expressed as fold change relative to the control group. Data are presented as mean ± SEM (n=5-8). No statistically significant differences were observed among the treatment groups (one-way ANOVA; p=0.1986).
