# Supplementary figures and images for: Taliglucerase Alfa Reduces Amyloid-β Burden by Restoring Autophagic Pathways in a Neuronal Model of Alzheimer’s Disease
Source: Neurochem Res. 2026 Jun 1;51(3):181. doi: 10.1007/s11064-026-04792-w (PMC13226380; doi:10.1007/s11064-026-04792-w)

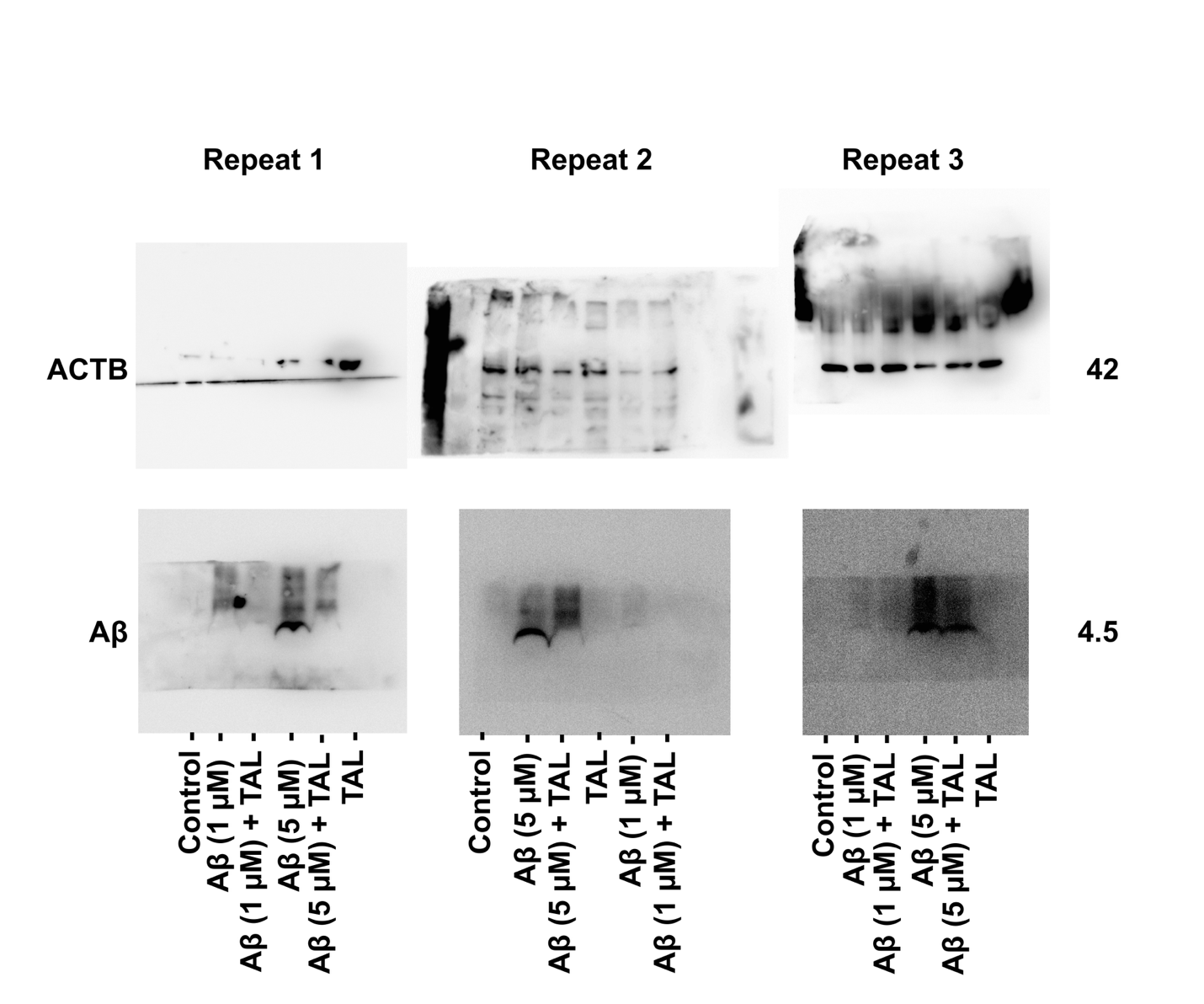


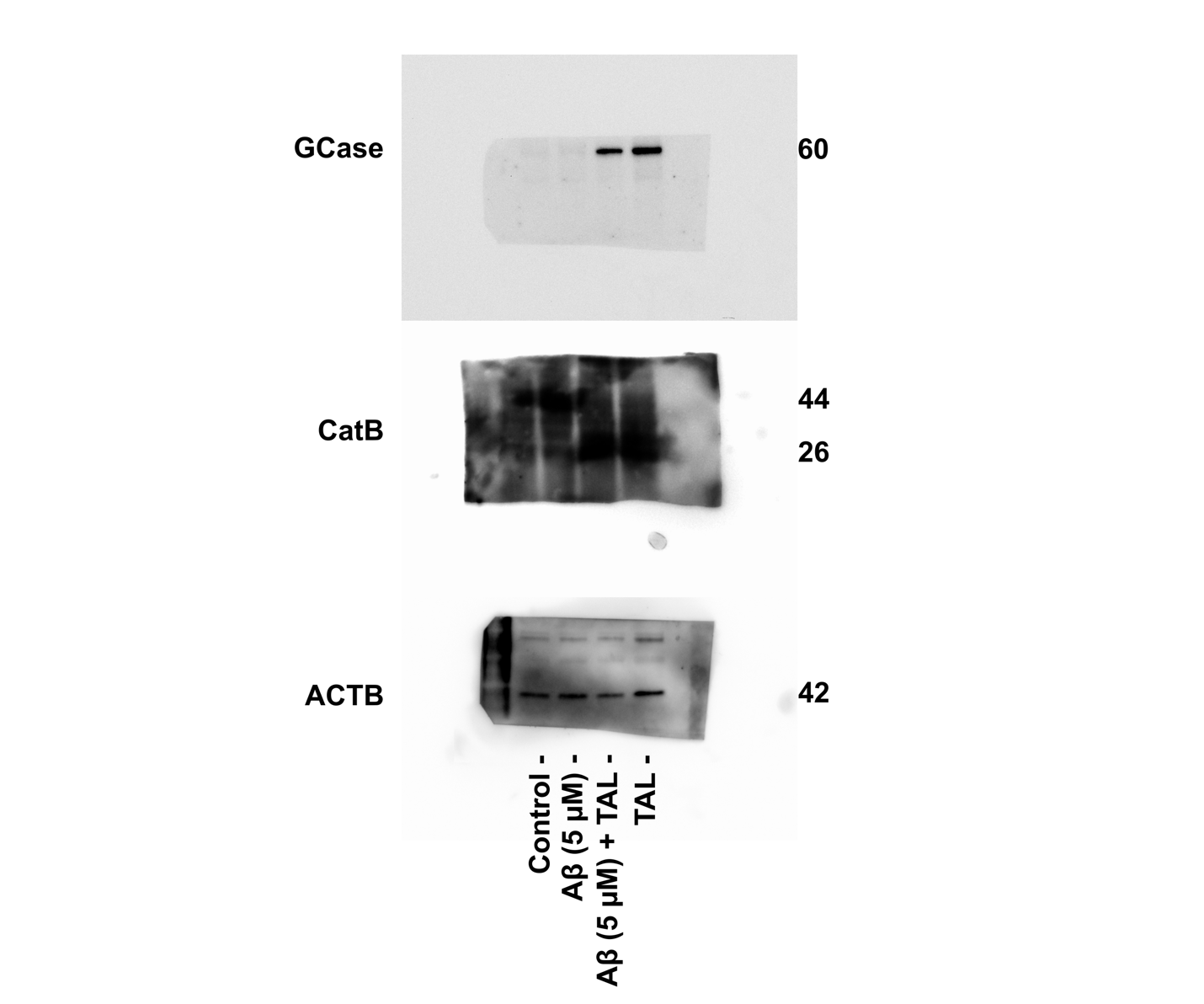

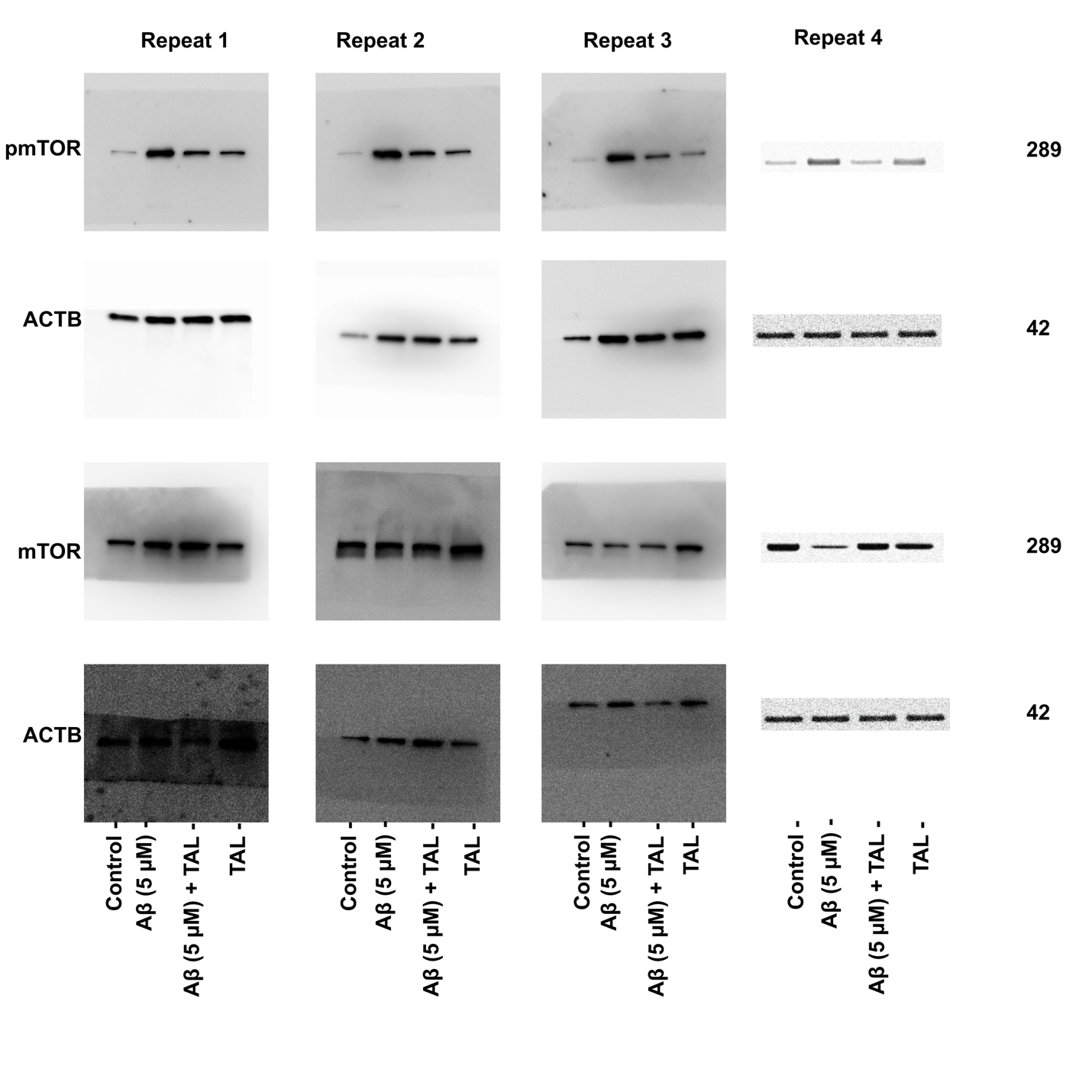

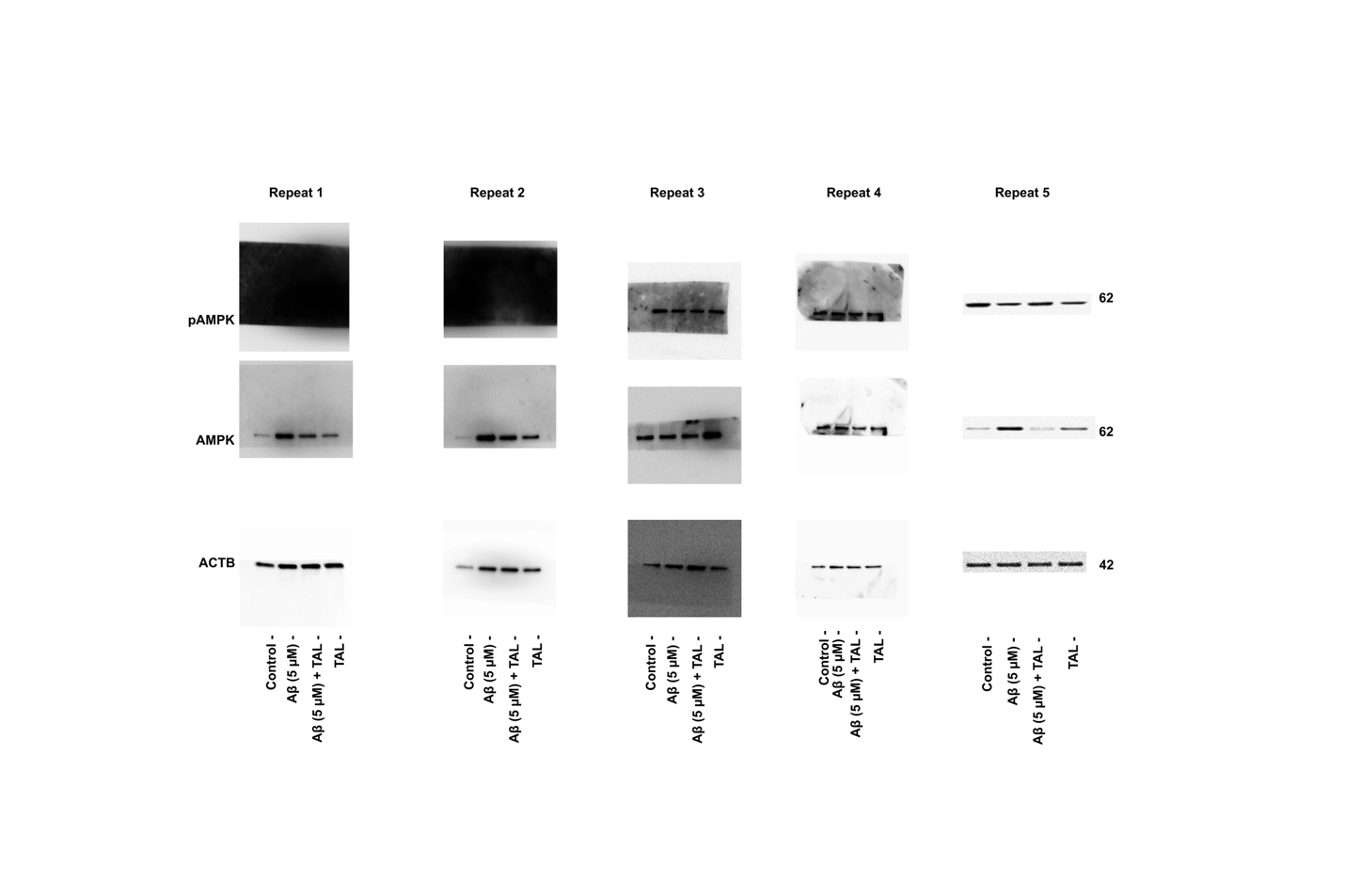

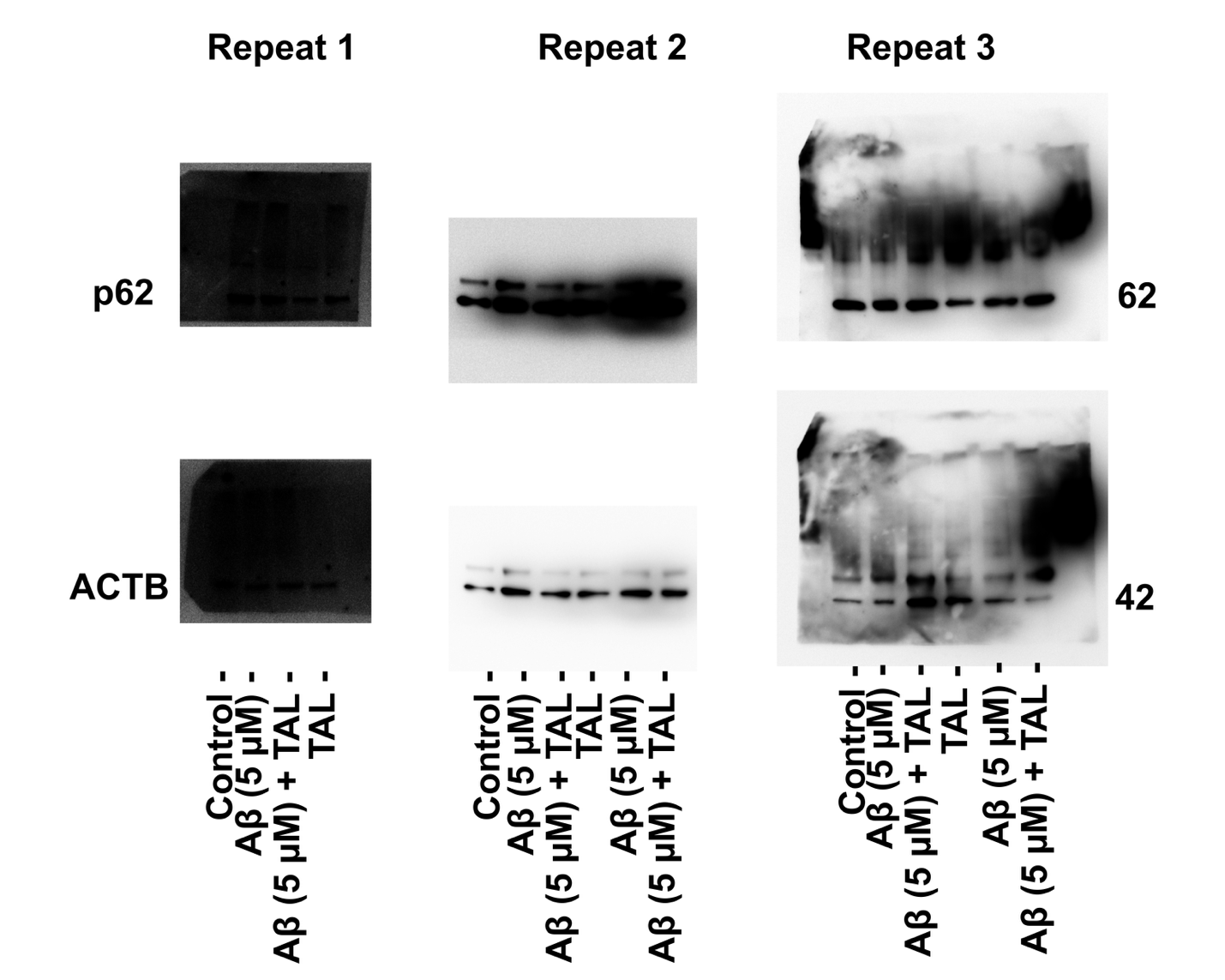

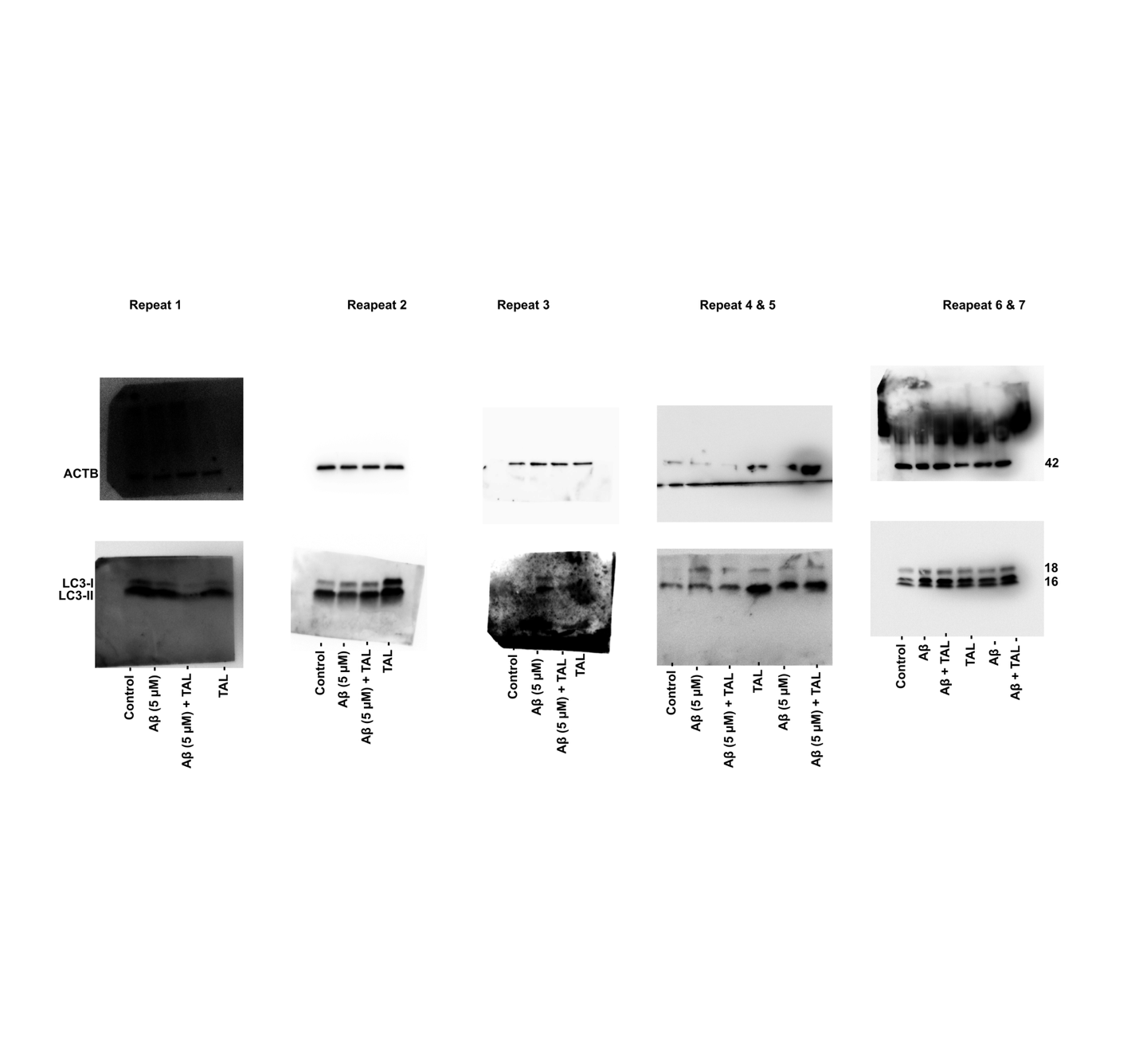

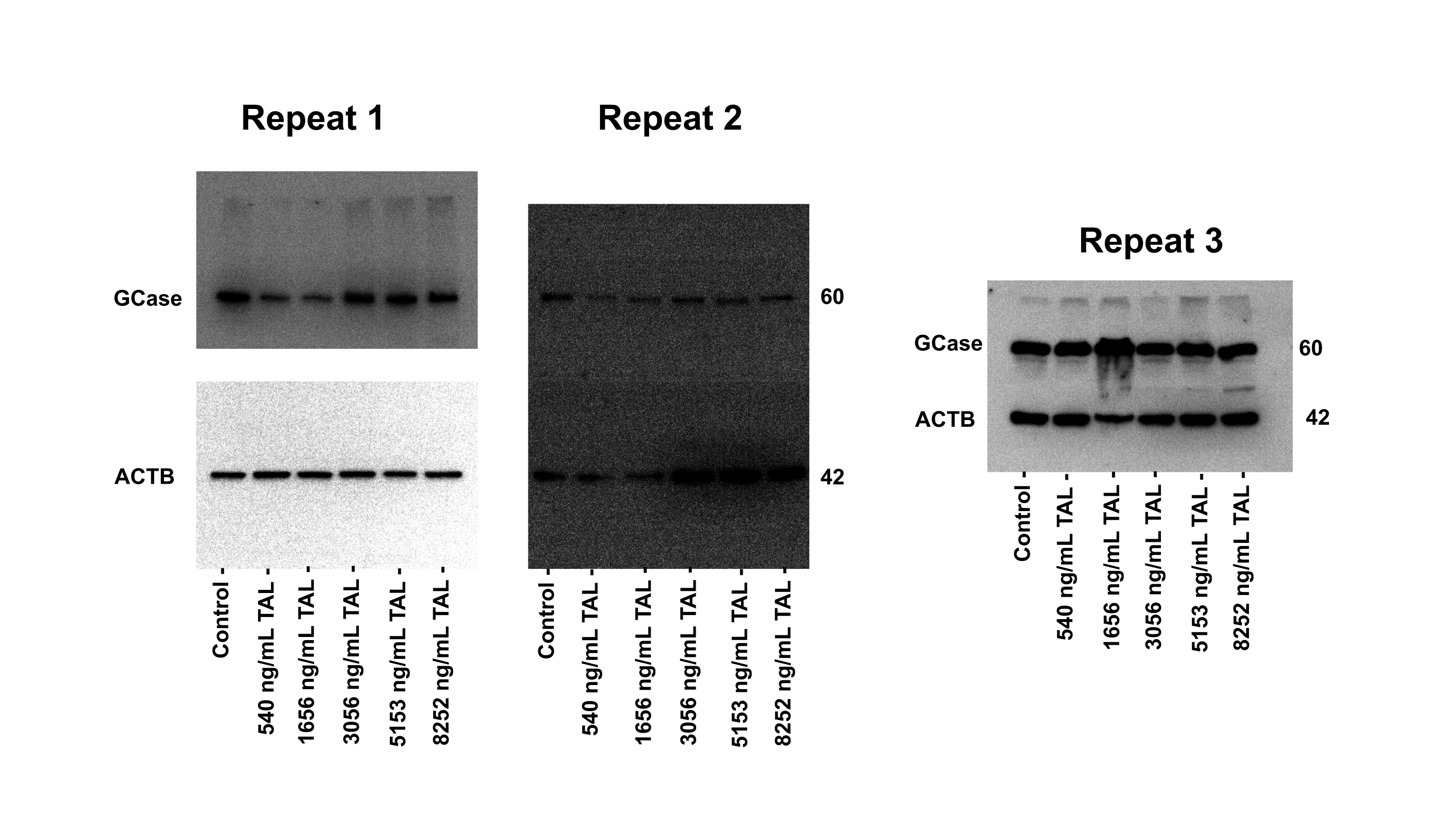

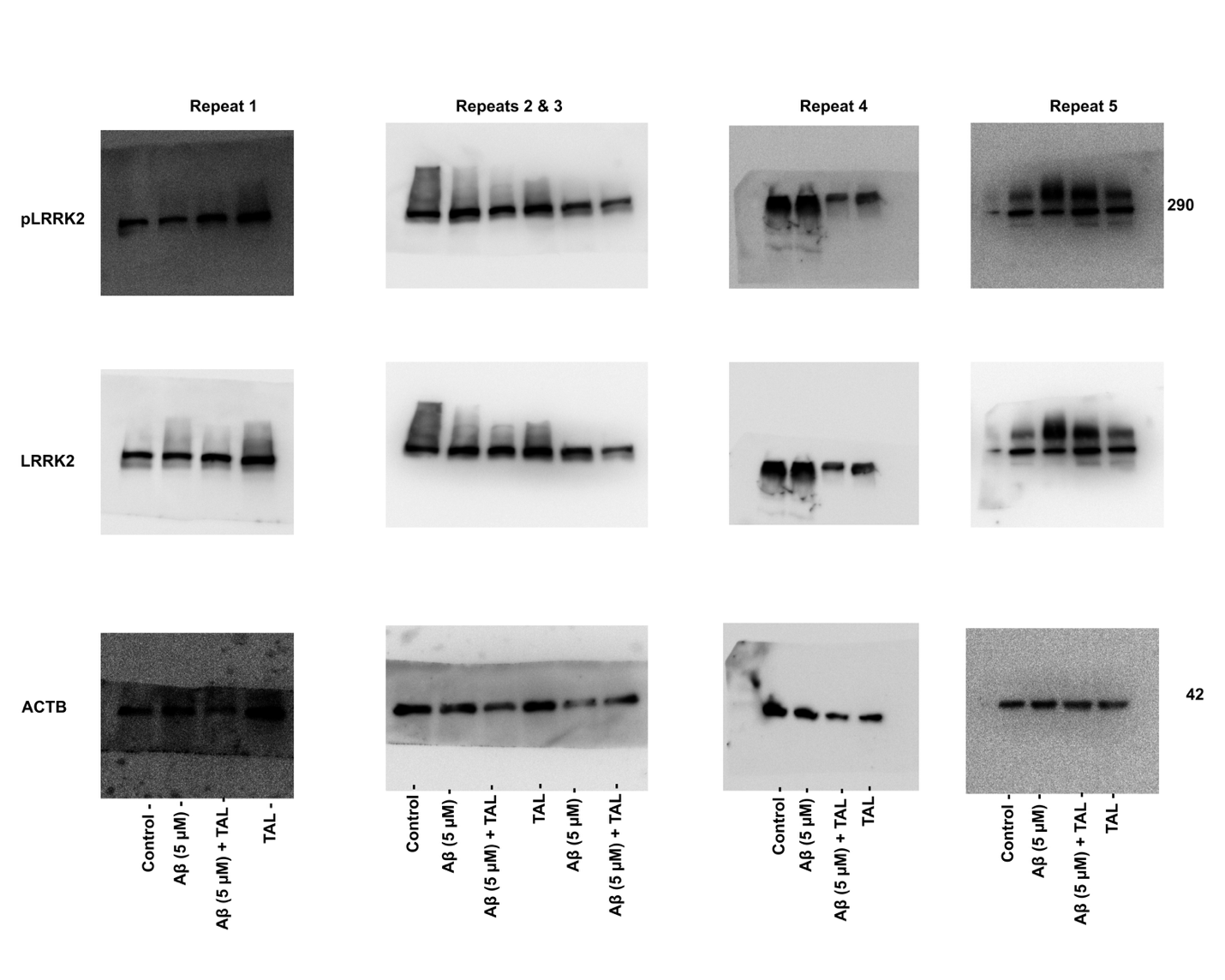

Supplement: Supplementary file 2 — Supplementary Material 2 [file 11064_2026_4792_MOESM2_ESM.docx]
